# The adaptation of the gut microbiome to social environmental changes in an Asian langur

**DOI:** 10.1016/j.isci.2026.116779

**Published:** 2026-07-21

**Authors:** Yanqiong Chen, Ying Lai, Zheng Liu, Kechu Zhang, Jingjin Zheng, Shiyi Lu, Zhonghao Huang

**Affiliations:** 1 Guangxi Key Laboratory of Biology for Mango, College of Agriculture and Food Engineering, Baise University, Baise, Guangxi 533000, China; 2 Key Laboratory of Ecology of Rare and Endangered Species and Environmental Protection (Guangxi Normal University), Ministry of Education, Guilin, Guangxi 541000, China; 3 The Chongzuo White-Headed Langur Field Observation and Research Station of Guangxi, Chongzuo, Guangxi 532200, China; 4 Institute of Life Sciences, Youjiang Medical University for Nationalities, Baise, Guangxi 533000, China; 5 Key Laboratory of Mountain Biodiversity Conservation, Education Department of Guangxi Zhuang Autonomous Region, Yulin Normal University, Yulin, Guangxi 537000, China; 6 Guangxi Forest Resources and Environment Monitoring Center, Nanning, Guangxi 530028, China

**Keywords:** gut microbiome, social environment, white-headed langur, Trachypithecus leucocephalus

## Abstract

Social environments profoundly impact social animals’ gut microbiome. Understanding such effects is critical for evaluating population fitness and conservation. Employing 16S rRNA and metagenomic sequencing, we investigated the gut microbiome of the endangered white-headed langur (*Trachypithecus leucocephalus*) to clarify its potential adaptive strategies to social environmental changes. Distinct differences were observed among social groups: the all-male group was enriched in Bacillota and showed stronger cellulose degradation potential, which might be associated with greater cellulose intake and higher cortisol and T3 levels; mixed-sex group was enriched in Actinomycetota, Pseudomonadota, and non-carbohydrate metabolism genes, possibly due to more young leaves consumption and reproductive needs. Alpha male replacement also shaped gut microbiome: the third alpha male period had highest Bacteroidota and lowest metabolic genes abundance, potentially related to improved food quality during this period. These preliminary findings highlight gut microbial adaptation to social environments in the studied population, providing implications for the conservation of this endangered species.

## Highlights

• Preliminary evidence of gut microbiome response to social environment in white-headed langurs
• All-male group is enriched in Bacillota and cellulose-degradation metabolism genes
• Mixed-sex group is enriched in Actinomycetota and non-carbohydrate metabolism genes
• Alpha male replacement alters the proportions of Bacteroidota and metabolic genes

## Graphical abstract

**Figure:**
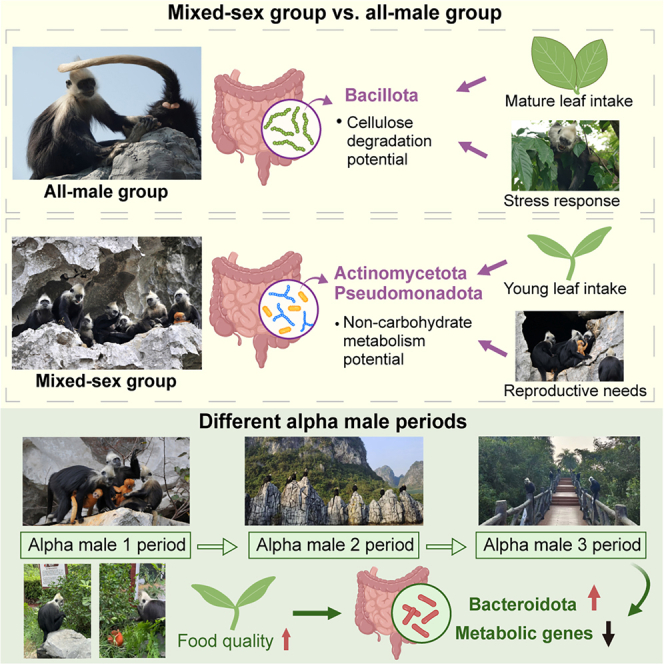

## Teaser

Nature conservation; Zoology; Microbiology; Microbial genomics

## Introduction

The gut microbiome benefits from the survival environment and nutrients provided by the host’s intestines, while playing a vital role in the animal’s metabolism, development, and immunity.^1^^,^^2^ According to metacommunity theory, host individuals are treated as “islands”. The formation of social groups facilitates the dispersal of gut microbiome between islands, resulting in the development of unique social microbiomes.^3^^,^^4^ However, social microbiome formation presents a dual nature for communities.^3^^,^^4^ On the one hand, the spread of beneficial taxa mediated by society contributes to enhanced stability and resilience within the group.^3^^,^^4^ On the other hand, the potential transmission of pathogens within the groups may severely compromise the health of community.^3^^,^^4^ Therefore, the gut microbiome may not only reshape the physiological characteristics and behavioral patterns of host populations, but also alter their evolutionary trajectories through long-term evolutionary processes, potentially emerging as a driving factor in collective adaptive evolution.^3^

The close relationship between the social environment and gut microbiome has been confirmed in multiple species including Kalahari meerkats (*Suricata suricatta*),^4^ yellow-bellied marmots (*Marmota flaviventer*),^5^ vampire bats (*Desmodus rotundus*),^6^ and ring-tailed lemurs (*Lemur catta*).^7^ The influence of social environments on gut microbiome mainly reflects three aspects: First, gut microbiome among individuals within the same group exhibit similarities.^8^^,^^9^ For example, closely distributed populations of the same species show significant intergroup differences in gut microbiome composition, while individuals from the same population maintain high similarity.^8^^,^^9^ Secondly, migrated individuals gradually develop a gut microbiome similar to that of the original members.^10^^,^^11^ The changes in community environment following immigration may also lead to significant dynamic shifts in the gut microbiome of the group.^10^^,^^11^ Finally, social behavior in animals not only impacts gut microbiome diversity and the transmission of beneficial bacteria, but also experiences a mutual influence from the gut microbiome in return.^3^^,^^8^ The association between social environments and gut microbiome offers another research perspective for elucidating the mechanisms of animal social ecology.

The characteristics of the gut microbiome in social groups are the result of the combined effects of multiple factors, of which shared food consumption and horizontal transmission between individuals exert the stronger influence.^8^^,^^12^^,^^13^ Food, a vital driver in the formation and stabilization of gut microbiome communities, exerts selective pressure on the gut microbiome by altering competitive relationships among bacteria.^1^^,^^14^ The homogenization of nutritional intake patterns in the same community leads to convergence in gut microbiome among its members.^8^^,^^12^ On the contrary, differences in dietary nutritional intake patterns may result in variations in the gut microbiome structures and functions between distinct groups.^8^^,^^12^ Additionally, social interactions (direct contact) or shared living environments (indirect contact) could facilitate horizontal transmission of gut microbiome.^13^ Direct contact between animals, such as grooming, maternal-infant carrying, and mating, could promote the spread of microbes between islands, with the intensity of this spread significantly influenced by the frequency of social interactions.^15^^,^^16^^,^^17^ Microorganisms in the substrates (e.g., water, soil) that group members come into contact with in their habitats may serve as sources for the gut microbiome.^13^ In conclusion, there are numerous factors in social living that are closely related to the gut microbiome. Given the vital role that the gut microbiome plays in animals, along with the transmissibility of bacteria within a population, studying how social environments influence gut microbiome is crucial for understanding group fitness and maintaining population health.

Hormones, as key physiological mediators of the host that influence the gut microbiome, are closely associated with animal sociality.^18^^,^^19^^,^^20^ Several factors in group living impose significant stress on animals, including competition for food, water, and mating rights, maintaining hierarchies within and between groups, and intergroup conflicts.^11^^,^^21^ Stress exposure affects the physiological state of the host, such as intestinal mucosal permeability and metabolic levels, by stimulating the animal’s hypothalamic-pituitary-adrenal (HPA) axis to release stress hormones, ultimately impacting the gut microbiome.^20^^,^^22^ For example, under prolonged exposure to environmental stressors (e.g., anthropogenic disturbance), the glucocorticoid (GC) level of black howler monkeys (*Alouatta pigra*) remains chronically elevated, which induces alterations in the diversity of their gut microbiota.^20^ In addition, hormones are also involved in regulating social behaviors of primates such as grooming, mating, and infant care.^18^^,^^19^ Changes in group nutritional stress, managing social relationships, and regulating social behavior also rely on hormones to adjust metabolic levels and provide energy.^23^^,^^24^ Thus, studying the relationship between group hormone levels and the social microbiome could serve as an avenue for understanding the adaptation of animals to diverse social environments.

The white-headed langur (*Trachypithecus leucocephalus*) is exclusively distributed in the karst limestone forest of Chongzuo, Guangxi, China, which is classified as a critically endangered (CR) species by the International Union for Conservation of Nature (IUCN).^25^^,^^26^ The social groups of the langurs are classified into two types, including mixed-sex groups and all-male groups.^25^^,^^27^ In mixed-sex groups, a sole adult male serves as the dominant leader and cohabits with the relevant females and infant langurs.^25^^,^^27^ The all-male group comprises adult males that have been expelled from mixed-sex groups.^25^^,^^27^ These males frequently engage in competitive challenges against the alpha male of a mixed-sex group in an attempt to assume leadership and thereby gain reproductive access to females.^27^^,^^28^ In behavioral ecology, marked differences exist between mixed-sex and all-male groups.^29^ Specifically, mixed-sex groups preferentially consume young leaves, whereas all-male groups rely more heavily on mature leaves; moreover, all-male groups exhibit larger home ranges and longer daily path lengths.^29^ The social structure of white-headed langurs is highly complex, and their social behavior patterns are exceptionally diverse, making them an ideal model species for exploring the links between social environment and their gut microbiome.

Previous studies have demonstrated that the gut microbiome of white-headed langurs is predominantly composed of Bacillota, an adaptation consistent with their highly folivorous diets.^30^^,^^31^^,^^32^ These studies have further delineated the temporal and spatial variation patterns of the gut microbiome.^30^^,^^31^^,^^32^ However, the changes in the gut microbiome of white-headed langurs in different social environments are still unknown, which is insufficient for us to understand the adaptation mechanism of these langurs to changes in social environments. Therefore, this study employed an integrated multi-omics approach, combining 16S rRNA and metagenomic sequencing to conduct a cross-sectional comparison of gut microbial communities between mixed-sex and all-male groups, and longitudinally assess the impact of alpha male replacement on the gut microbiome. Concurrently, physiological hormone analyses are incorporated to further elucidate how social environmental factors modulate the gut microbiome. The aim of this study is to reveal the adaptation mechanisms of the gut microbiome of white-headed langurs to social environment changes in the fragmented habitats, providing a scientific basis for the conservation of this species. The study tests the following predictions:

### Prediction 1

Behavioral patterns differ between mixed-sex and all-male groups.^29^ We predict that the structures and functions of the gut microbiome in these two social types also have significant differences: The all-male group, which feeds on more mature leaves, may enrich more bacteria and functional genes related to dietary fiber degradation; whereas the mixed-sex group, as a reproductive group, may contain more bacteria and functional genes related to reproductive activities.^29^

### Prediction 2

Variations in social stress levels and reproductive status across different social environments are likely to influence the gut microbiome by regulating hormone release.^18^^,^^20^ Therefore, we predict that the all-male group, which faces the pressure of competing for reproductive rights, may display functional genes related to polysaccharide metabolism and energy production more than the mixed-sex group, potentially in association with higher stress hormone secretion levels.

### Prediction 3

Alpha male replacement involves substantial reorganization of social networks and shifts in the broader social environment, which may be accompanied by corresponding changes in the gut microbiome.^11^^,^^33^ Therefore, we predict that the diversity, composition, and functions of the gut microbiome of the langur groups may show significant differences during different male dominance periods.

### Prediction 4

During alpha male replacement, exposure to the stress of group instability and infanticide risk may lead to the release of stress hormones, which could be linked to changes in the gut microbiome.^11^ We predict that the relative abundances of bacteria or metabolic genes related to short-chain fatty acid (SCFAs) metabolism may increase, potentially in response to increased stress and energy deficiency.

## Results

### The gut microbiome of the white-headed langurs

All samples were clustered and generated 276,086 amplicon sequence variants (ASVs). Detailed information on 16S rRNA sequencing was shown in Table S1. The gut microbiome of white-headed langurs was dominated by Bacillota (83.84% ± 14.75%), Pseudomonadota (5.28% ± 12.62%), and Actinomycetota (4.65% ± 5.71%) at the phylum level. At the family level, it was mainly composed of Oscillospiraceae (15.56% ± 5.51%), norank_o__Clostridia_UCG-014 (14.66% ± 6.99%), and Christensenellaceae (13.87% ± 5.95%). Functionally, the gut microbiome of the langurs mainly performed the Metabolism function (47.67% ± 1.92%), followed by Genetic Information Processing (17.62% ± 2.28%) and Environmental Information Processing (14.33% ± 0.67%). Regarding carbohydrate-active enzymes (CAZy), the gut microbiome mainly synthesized Glycoside Hydrolases (42.37% ± 5.03%), Glycosyl Transferases (34.36% ± 2.96%), and Carbohydrate Esterases (15.75% ± 2.59%). Relative abundances of other microbial and functional genes for each white-headed langur gut microbiome sample were shown in Table S2.

### The comparison of gut microbiome between the mixed-sex group and all-male group

#### The comparison of gut microbiome structure

**Figure 1:**
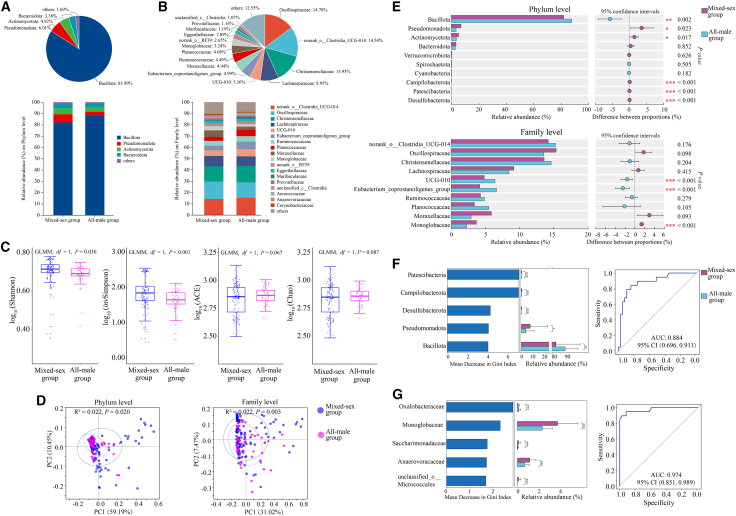
Comparison of gut microbiome structure between the mixed-sex group and all-male group (A) Relative abundances of gut microbiome at the phylum level in mixed-sex and all-male groups. “Others” include all taxa whose relative abundance is less than 1%. (B) Relative abundances of gut microbiome at the family level in mixed-sex and all-male groups. “Others” include all taxa whose relative abundance is less than 1%. (C) Comparison of alpha diversity indices based on GLMM. Data are represented as mean ± SD. (D) Results of PCoA in phylum and family levels. (E) Comparison of dominant taxa based on GLMM. (F) Results of RF at the phylum level. Data are represented as mean ± SD. (G) Results of RF at the family level. Data are represented as mean ± SD. ∗ for *p* < 0.05, ∗∗ for *p* < 0.01, and ∗∗∗ for *p* < 0.001.

The mixed-sex group of white-headed langurs clustered into 31,390 ASVs, annotated into 30 phyla and 359 families; the all-male group clustered into 12,794 ASVs, annotated into 22 phyla and 243 families. Both social groups at the phylum level were dominated by Bacillota (mixed-sex group: 82.11% ± 15.39%; all-male group: 87.94% ± 8.25%) (Figure 1A). At the family level, the mixed-sex group showed the highest relative abundance in Oscillospiraceae (15.36% ± 5.91%), while the all-male group was the most abundant in norank_o__Clostridia_UCG-014 (15.27% ± 6.35%) (Figure 1B).

The diversity indices of the mixed-sex group were significantly higher than that of the all-male group (Shannon index: 5.14 ± 0.66 vs. 4.88 ± 0.51, *χ*^*2*^ = 4.409, *df* = 1, *p* = 0.036; *inv*Simpson index: 112.32 ± 104.68 vs. 52.34 ± 28.73, *χ*^*2*^ = 12.897, *df* = 1, *p* < 0.001), whereas there were no significant difference in the richness (ACE, Chao) indices (Figure 1C). The details of generalized linear mixed models (GLMM) were provided in Table S3. The principal coordinates analysis (PCoA) analysis revealed significant differences in the community structure of the gut microbiome between the mixed-sex group and the all-male group (*p* < 0.05) (Figure 1D).

The results of GLMM indicated that at the phylum level, the relative abundances of Pseudomonadota and Actinomycetota were significantly higher in the mixed-sex group than those in the all-male group; conversely, the proportion of Bacillota was markedly more abundant in the all-male group than the mixed-sex group (Figure 1E). At the family level, Monoglobaceae was significantly more abundant in the mixed-sex group than in the all-male group, while the proportions of UCG-010 and Eubacterium_coprostanoligenes_group were significantly higher in the all-male group (Figure 1E). Other microbial comparisons based on GLMM were presented in Table S3. The random forest (RF) analysis revealed Patescibacteria as the most significant contributor to gut microbiome structural differences between the mixed-sex group and the all-male group at the phylum level (Figure 1F), whereas Oxalobacteraceae exhibited the highest contribution at the family level (Figure 1G).

#### The comparison of gut microbiome functional genes

**Figure 2:**
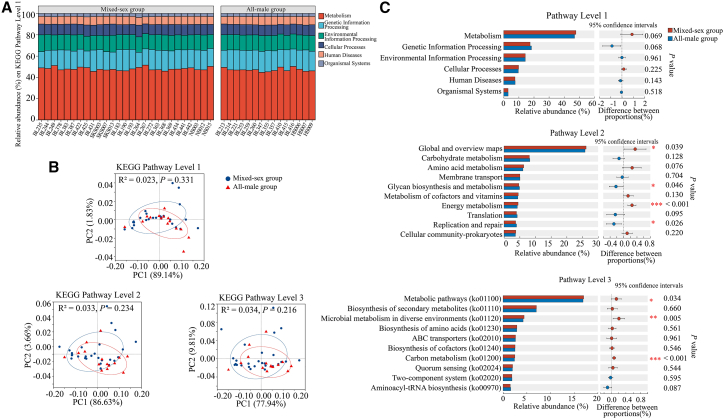
Comparison of functional genes of the gut microbiome between the mixed-sex group and all-male group (A) Relative abundances of the gut microbiome KEGG Level 1 pathways in mixed-sex and all-male groups. (B) Results of PCoA in Level 1 to 3 pathways. (C) Comparison of KEGG pathways genes based on GLMM. ∗ for *p* < 0.05, ∗∗ for *p* < 0.01, and ∗∗∗ for *p* < 0.001.

According to the Kyoto Encyclopedia of Genes and Genomes (KEGG) pathways, the relative abundance of the Metabolism gene was the highest both in the mixed-sex (47.85% ± 1.50%) and all-male groups (46.96% ± 1.59%) (Figure 2A). Global and overview maps, carbohydrate metabolism, and amino acid metabolism were the most abundant genes under the Metabolism pathway, accounting for 26.20% ± 0.53%, 8.10% ± 0.26%, and 6.30% ± 0.46% respectively in the mixed-sex group; and 25.85% ± 0.54%, 8.23% ± 0.28%, and 6.04% ± 0.47% respectively in the all-male group. The relative abundances of KEGG functional genes were shown in Table S4.

The PCoA results showed that there were no significant differences in functional genes at the different levels between the mixed-sex group and the all-male group (Figure 2B). The GLMM results indicated that the mixed-sex group enriched more Level 2 pathways such as global and overview maps, energy metabolism, metabolism of other amino acids, and Level 3 pathways including ko01100 (Metabolic pathways), ko01120 (Microbial metabolism in diverse environments) than in the all-male group (Figure 2C). Meanwhile, the proportions of glycan biosynthesis and metabolism, replication and repair (Level 2), and ko03440 (Homologous recombination), ko00520 (Amino sugar and nucleotide sugar metabolism) (Level 3) were significantly higher in the all-male group than in the mixed-sex group (Figure 2C). Other comparisons of KEGG functional genes based on GLMM were presented in Table S5.

Additionally, the CAZy annotation revealed that both the mixed-sex group and all-male group were enriched with Glycoside Hydrolases (mixed-sex group: 41.30% ± 3.41%, all-male group: 42.75% ± 2.81%) and Glycosyl Transferases genes (mixed-sex group: 34.93% ± 1.96%, all-male group: 35.16% ± 2.52%) (Figure S1A). PCoA results indicated that there were no significant differences in CAZy functional genes between the mixed-sex group and all-male group (Figure S1B). The GLMM found that the relative abundances of Auxiliary Activities (Class level), CE10 and AA3 (Family level) were significantly higher in the mixed-sex group than in the all-male group, while the all-male group enriched more GT2_Glycos_transf_2 and GT8 genes than the mixed-sex group (Figure S1C). Other comparisons of CAZy functional genes based on GLMM were presented in Table S4.

In summary, we summarized the key microbial taxa and functional genes that differed in the gut microbiome between the mixed-sex group and the all-male group in the Table 1.

**Table 1:** The key microbial and potential functional differences between mixed-sex and all-male groups

Level	Key variables	Relative abundance (Mean ± SD)		*χ*^*2*^ (*df* = 1)	*p* value
		Mixed-sex group	All-male group		
Gut microbiome structure	Shannon index	5.14 ± 0.66	4.88 ± 0.51	4.409	0.036
	*inv*Simpson index	112.32 ± 104.68	52.34 ± 28.73	12.897	<0.001
	Bacillota	82.11% ± 15.39%	87.94% ± 8.25%	9.465	0.002
	Pseudomonadota	7.18% ± 14.31%	3.55% ± 6.91%	5.149	0.023
	Actinomycetota	5.41% ± 5.30%	3.90% ± 3.39%	5.714	0.017
	UCG-010	4.86% ± 3.63%	6.41% ± 4.12%	11.681	<0.001
	Eubacterium_coprostanoligenes_group	4.20% ± 2.19%	6.64% ± 5.46%	19.016	<0.001
	Monoglobaceae	3.70% ± 1.88%	2.29% ± 0.93%	26.269	<0.001
Gut microbiome functional genes	Metabolism	47.85% ± 1.50%	46.96% ± 1.59%	3.297	0.069
	Global and overview maps	26.2% ± 0.53%	25.85% ± 0.54%	4.252	0.039
	Glycan biosynthesis and metabolism	4.73% ± 0.42%	4.96% ± 0.29%	3.980	0.046
	Energy metabolism	4.50% ± 0.18%	4.24% ± 0.21%	16.203	<0.001
	Metabolism of other amino acids	1.99% ± 0.13%	1.88% ± 0.09%	7.572	0.006
	Metabolism of terpenoids and polyketides	1.30% ± 0.08%	1.25% ± 0.06%	4.097	0.043
	Metabolic pathways (ko01100)	17.02% ± 0.20%	16.88% ± 0.21%	4.470	0.034
	Carbon metabolism (ko01200)	2.49% ± 0.08%	2.42% ± 0.07%	11.898	<0.001
	Amino sugar and nucleotide sugar metabolism (ko00520)	1.21% ± 0.09%	1.28% ± 0.06%	6.783	0.009
	Auxiliary activities	4.57% ± 1.36%	3.80% ± 0.84%	4.729	0.030
	GT2_Glycos_transf_2	10.50% ± 1.01%	11.12% ± 1.05%	4.906	0.027
	CE10	3.74% ± 0.88%	3.22% ± 0.73%	3.990	0.046

#### The correlations between hormones and the gut microbiome

The GLMM results showed that the levels of GC (64.65 ± 8.02 μg/g vs. 76.54 ± 3.91 μg/g, *χ*^*2*^ = 8.210, *df* = 1, *p* = 0.004) and T3 (18.16 ± 8.02 ng/g vs. 19.35 ± 0.94 ng/g, *χ*^*2*^ = 7.405, *df* = 1, *p* = 0.007) in mixed-sex group were significantly lower than that in all-male group; while there were no significant differences in cortisol (9.88 ± 1.09 μg/g vs. 10.57 ± 0.72 μg/g, *χ*^*2*^ = 1.481, *df* = 1, *p* = 0.224) and T4 levels (36.25 ± 4.98 ng/g vs. 39.39 ± 3.04 ng/g, *χ*^*2*^ = 1.385, *df* = 1, *p* = 0.239) (Figure S2).

**Figure 3:**
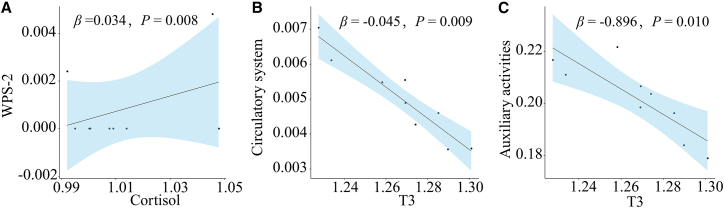
The correlations between hormones and the gut microbiome in mixed-sex and all-male groups based on the GLM Only results with *p* ≤ 0.01 are presented here. (A) Correlation between cortisol and WPS-2. (B) Correlation between T3 and Circulatory system. (C) Correlation between T3 and Auxiliary activities. The Y-axis represents the relative abundance of microbial taxa (arcsine square-root transformation), and the X-axis represents hormone levels (log_10_(x) transformation).

The generalized linear model (GLM) results showed the associations of hormones and the gut microbiome. At the phylum level, the cortisol level was significantly positively correlated with the relative abundance of Bacillota (*β* = 2.766, *p* = 0.043) (Figure S3A) and WPS-2 (*β* = 0.034, *p* = 0.008) (Figure 3A). At the family level, the proportion of Eubacterium_coprostanoligenes_group showed a significant positive correlation with cortisol (*β* = 2.476, *p* = 0.038) (Figure S3E); Ruminococcaceae was significantly positively correlated with T3 (*β* = 2.308, *p* = 0.027) (Figure S3F); and GC level showed a significant positive correlation with the proportion of Aerococcaceae (*β* = 2.020, *p* = 0.033) (Figure S3I).

Functionally, the proportion of Cellular Processes (Level 1, *β* = −0.175, *p* = 0.014) (Figure S4A) showed a significant negative correlation with cortisol. The T3 level was significantly positively correlated with glycan biosynthesis and metabolism (Level 2, *β* = 0.174, *p* = 0.035) (Figure S4B), drug resistance: antimicrobial (Level 2, *β* = 0.201, *p* = 0.026) (Figure S4F) and ko00520 (Level 3, *β* = 0.076, *p* = 0.034) (Figure S4I), and negatively correlated with circulatory system (Level 2, *β* = −0.045, *p* = 0.009 (Figure 3B). The T4 level showed a significant positive correlation with the proportion of folding sorting and degradation (Level 2, *β* = 0.063, *p* = 0.024) (Figure S4D) and negatively correlated with nervous system (Level 2, *β* = −0.026, *p* = 0.023) (Figure S4G). In the CAZy function, the Auxiliary Activities were significantly negatively correlated with T3 (*β* = −0.896, *p* = 0.010) (Figure 3C). At the family level, the GT4 was significantly negatively correlated with cortisol (*β* = −0.364, *p* = 0.033) (Figure S5B); the GT41 was markedly negatively correlated with GC (*β* = −0.249, *p* = 0.017) (Figure S5E) and T4 (*β* = −0.123, *p* = 0.042) (Figure S5G), and was positively correlated with T3 (*β* = 0.433, *p* = 0.028) (Figure S5F). Additional correlations of microbial taxa or functional genes with hormones based on GLM were shown in Table S6.

### The comparison of the gut microbiome of the different male periods

#### The comparison of gut microbiome structure

**Figure 4:**
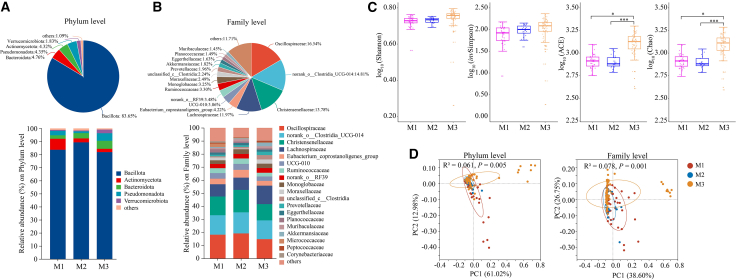
Comparison of gut microbiome structure in different alpha male periods (A) Relative abundances of the gut microbiome at the phylum level in different alpha male periods. “Others” include all taxa whose relative abundance is less than 1%. (B) Relative abundances of the gut microbiome at the family level in different alpha male periods. “Others” include all taxa whose relative abundance is less than 1%. (C) Comparison of alpha diversity indices based on GLMM. Data are represented as mean ± SD. (D) Results of the PCoA. M1: the period dominated by alpha male 1, M2: the period dominated by alpha male 2, M3: the period dominated by alpha male 3. ∗ for *p* < 0.05, ∗∗ for *p* < 0.01, and ∗∗∗ for *p* < 0.001.

There are three alpha male tenure periods: The period dominated by alpha male 1 is defined as M1; the period dominated by alpha male 2 as M2; and the period dominated by alpha male 3 as M3. The M1 samples clustered into 9,288 ASVs, with taxonomic annotations assigned to 27 phyla and 308 families; while M2 and M3 samples clustered into 5,536 and 61,465 ASVs, with taxonomic annotations for 26 and 30 phyla and 326, 306 families, respectively. In different periods, the gut microbiome of the langurs was dominated by Bacillota (M1: 83.68% ± 12.79%; M2: 89.34% ± 4.49%; M3: 81.86% ± 19.23%) (Figure 4A); at the family level, Oscillospiraceae had the highest proportion (M1: 18.22% ± 4.76%; M2: 19.18% ± 3.45%; M3: 14.80% ± 5.26%) (Figure 4B).

After controlling for seasonal effects using GLMMs, no significant differences were observed in Shannon or *inv*Simpson indices between different alpha male periods, However, ACE (M1 vs. M3: 826.89 ± 147.18 vs. 1310.83 ± 345.07, *χ*^*2*^ = 6.074, *df* = 1, *p* = 0.014; M2 vs. M3: 805.94 ± 132.40 vs. 1310.83 ± 345.07, *χ*^*2*^ = 34.282, *df* = 1, *p* < 0.001) and Chao indices (M1 vs. M3: 822.54 ± 145.98 vs. 1270.34 ± 328.52, *χ*^*2*^ = 5.878, *df* = 1, *p* = 0.015; M2 vs. M3: 800.28 ± 127.90 vs. 1270.34 ± 328.52, *χ*^*2*^ = 32.593, *df* = 1, *p* < 0.001) were significantly higher in M3 than in M1 and M2 (Figure 4C). The details of the GLMM were provided in Table S7. The PCoA analysis showed that there were significant differences in the gut microbiome structure in different alpha male periods (*p* < 0.01) (Figure 4D).

**Figure 5:**
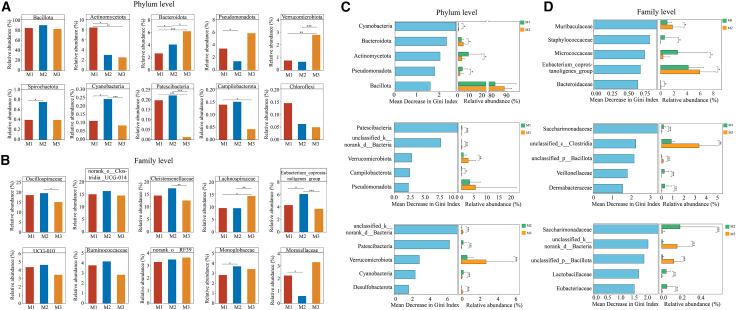
Comparison of dominant taxa in different alpha male periods (A) Comparison of dominant taxa at the phylum level based on GLMM. (B) Comparison of dominant taxa at the family level based on GLMM. (C) Results of RF at the phylum level. (D) Results of RF at the family level. M1: the period dominated by alpha male 1, M2: the period dominated by alpha male 2, M3: the period dominated by alpha male 3. ∗ for *p* < 0.05, ∗∗ for *p* < 0.01, and ∗∗∗ for *p* < 0.001.

Notably, the proportion of Actinomycetota in M1 was significantly higher than in M2 and M3; whereas Bacteroidota showed a markedly higher proportion in M3 compared to M1 and M2, with a significantly higher proportion in M2 than in M1 (Figure 5A). At the family level, the relative abundances of Oscillospiraceae and Christensenellaceae were significantly higher in M2 than in M3; the proportion of Lachnospiraceae was markedly higher in M3 than in M1 and M2; and the abundance of Eubacterium_coprostanoligenes_group was significantly higher in M2 than in M1 and M3 (Figure 5B). Other microbial comparisons based on GLMM and K-W test were presented in Table S7. In addition, the results of RF demonstrated that cyanobacteria contributed most to the M1 vs. M2 difference; Patescibacteria had the highest differential contribution in M1 vs. M3; and unclassified_k__norank_d__Bacteria was the primary contributor to the difference in M2 vs. M3 (Figure 5C). Additionally, the most contributory taxa at the family level were Muribaculaceae (M1 vs. M2) and Saccharimonadaceae (M1 vs. M3; M2 vs. M3) (Figure 5D).

#### The comparison of gut microbiome functional genes

**Figure 6:**
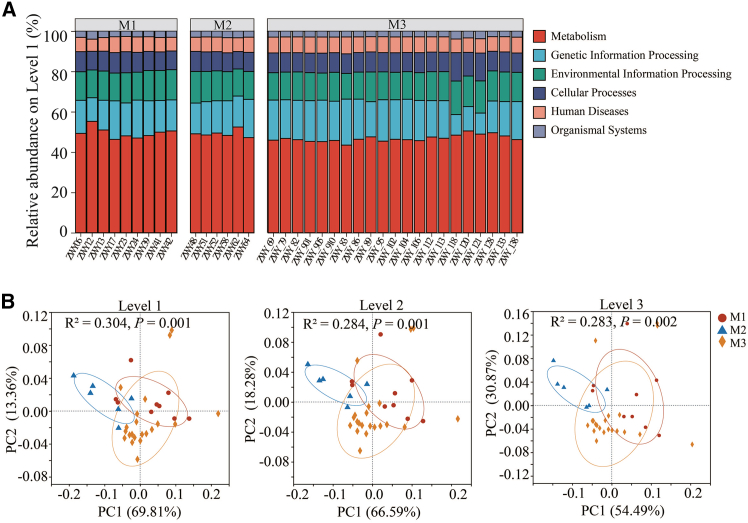
Comparison of functional genes of the gut microbiome in different alpha male periods (A) Relative abundances of the gut microbiome KEGG Level 1 pathways in different alpha male periods. (B) Results of the PCoA in Level 1 to 3 pathways. M1: the period dominated by alpha male 1, M2: the period dominated by alpha male 2, M3: the period dominated by alpha male 3. ∗ for *p* < 0.05, ∗∗ for *p* < 0.01, and ∗∗∗ for *p* < 0.001.

**Figure 7:**
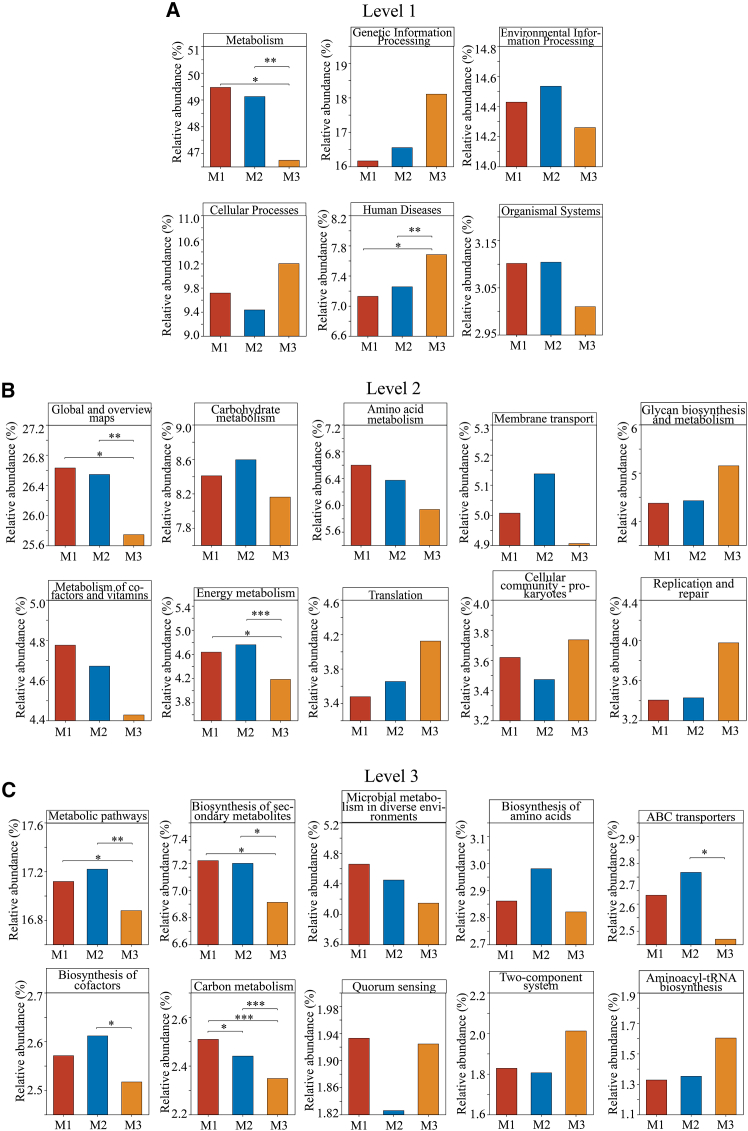
Comparison of KEGG pathways genes in different alpha male periods based on GLMM (A) Comparison of KEGG Level 1 pathways genes. (B) Comparison of KEGG Level 2 pathways genes. (C) Comparison of KEGG Level 3 pathways genes. M1: the period dominated by alpha male 1, M2: the period dominated by alpha male 2, M3: the period dominated by alpha male 3. ∗ for *p* < 0.05, ∗∗ for *p* < 0.01, and ∗∗∗ for *p* < 0.001.

During different alpha male periods, the gut microbiome of white-headed langurs primarily performed Metabolism (Level 1) functions (M1: 49.46% ± 2.66%; M2: 49.12% ± 1.79%; M3: 46.74% ± 1.63%) (Figure 6A). Under this pathway, the most prevalent Level 2 and Level 3 pathways were global and overview maps and metabolic pathways, respectively. The PCoA results indicated that, at different levels, the functional genes of the gut microbiome in the langurs showed significant differences in the three different alpha male periods (Figure 6B). We performed multiple comparisons of the relative abundances of functional genes based on the Kruskal-Wallis (K-W) test, with the results presented in Table S8. Additionally, we performed GLMM to compare differences between every two periods. The results showed that at Level 1, the relative abundance of Metabolism in M3 was significantly lower than that in M1 and M2, while the proportion of Human Diseases was significantly higher than that in M1 and M2 (Figure 7A). At Level 2, the relatively lower abundance of pathways such as global and overview maps, and energy metabolism in M3 compared to M1 and M2 was observed (Figure 7B). At Level 3, the proportions of ko01100 (Metabolic pathways), ko01110 (Biosynthesis of secondary metabolites) in M3 were significantly lower than those in the previous two periods (Figure 7C). Comparisons of other KEGG functional genes based on GLMM were shown in Table S8.

In addition, Glycoside Hydrolases exhibited the highest proportion in all three main stages (M1: 40.03% ± 5.36%; M2: 44.60% ± 3.93%; M3: 43.84% ± 7.40%) (Figure S6A). The PCoA indicated significant differences in CAZy functional genes among different alpha male periods at both the Class and Family levels (Figure S6B). GLMM results revealed that the significantly higher relative abundance of Polysaccharide Lyases in M3 than that in M1 and M2, while Cellulosome Modules showed a higher proportion in M2 and M3 compared to M1 (Figure S6C). At the family level, the proportion of GT2_Glycos_transf_2 and CE10 in M2 were significantly higher than in M3; and M3 enriched more GH3 genes than the previous two periods (Figure S6D). Other CAZy gene comparisons based on GLMM and K-W test were presented in Table S9.

In summary, we identified the key microbial taxa and functional genes that differed in the gut microbiome among different alpha male periods, as summarized in Table 2.

**Table 2:** The key microbial and potential functional differences in different alpha male periods (M1: the period dominated by alpha male 1, M2: the period dominated by alpha male 2, M3: the period dominated by alpha male 3)

Level	Key variables	Relative abundance (Mean ± SD)			*P-*(M1 vs. M2)	*P*-(M1 vs. M3)	*P*-(M2 vs. M3)
		M1	M2	M3			
Gut microbiome structure	ACE index	826.89 ± 147.18	805.94 ± 132.40	1310.83 ± 345.07	0.581	0.014	<0.001
	Chao index	822.54 ± 145.98	800.28 ± 127.90	1270.34 ± 328.52	0.558	0.015	<0.001
	Actinomycetota	8.40% ± 11.00%	3.00% ± 1.85%	2.53% ± 2.25%	0.028	0.001	0.160
	Bacteroidota	2.64% ± 1.48%	4.04% ± 1.78%	6.13% ± 4.20%	0.017	<0.001	0.016
	Eubacterium_coprostanoligenes_group	4.23% ± 2.37%	5.98% ± 2.36%	3.66% ± 2.48%	0.023	0.266	<0.001
Gut microbiome functional genes	Metabolism	49.46% ± 2.66%	49.12% ± 1.79%	46.74% ± 1.63%	0.769	0.040	0.004
	Human Diseases	7.13% ± 0.55%	7.26% ± 0.29%	7.68% ± 0.36%	0.570	0.044	0.005
	Global and overview maps	26.63% ± 0.74%	26.54% ± 0.55%	25.74% ± 0.56%	0.795	0.044	0.004
	Energy metabolism	4.64% ± 0.24%	4.76% ± 0.29%	4.18% ± 0.25%	0.351	0.015	<0.001
	Metabolism of other amino acids	2.11% ± 0.17%	2.06% ± 0.09%	1.83% ± 0.20%	0.510	0.023	0.009
	Metabolic pathways (ko01100)	17.12% ± 0.15%	17.22% ± 0.17%	16.88% ± 0.24%	0.204	0.047	0.006
	Biosynthesis of secondary metabolites (ko01110)	7.22% ± 0.18%	7.20% ± 0.22%	6.91% ± 0.21%	0.833	0.020	0.018
	Carbon metabolism (ko01200)	2.51% ± 0.06%	2.44% ± 0.04%	2.35% ± 0.04%	0.037	<0.001	<0.001
	GH3	2.51% ± 0.60%	2.45% ± 0.33%	1.78% ± 0.18%	0.870	0.002	<0.001
	GH78	1.79% ± 0.84%	2.77% ± 0.44%	1.72% ± 0.62%	0.034	0.833	0.010

#### Correlation between hormones and gut microbiome

In M1, langurs exhibited GC, cortisol, T3, and T4 levels of 81.41 ± 4.50 μg/g, 10.04 ± 0.63 μg/g, 18.71 ± 1.21 ng/g, and 36.89 ± 2.05 ng/g, respectively. In M2, the level of GC was 69.98 ± 6.85 μg/g, cortisol was 10.65 ± 0.88 μg/g, T3 was 19.55 ± 1.60 ng/g, and T4 was 37.20 ± 2.91 ng/g. The levels of GC, cortisol, T3, and T4 in M3 were 71.82 ± 5.82 μg/g, 10.38 ± 0.93 μg/g, 19.69 ± 1.56 ng/g and 38.17 ± 3.50 ng/g, respectively, in M3. The GLMM results showed that the GC level of M1 was significantly higher than that of M2 (*χ*^*2*^ = 6.329, *df* = 1, *p* = 0.012) and M3 (*χ*^*2*^ = 11.932, *df* = 1, *p* = 0.001). Cortisol was significantly higher in M2 than in M1 (*χ*^*2*^ = 6.359, *df* = 1, *p* = 0.012). T3 level was significantly greater in M3 than in M1 (*χ*^*2*^ = 5.372, *df* = 1, *p* = 0.020), and T4 level was significantly higher in M3 compared with both M1 (*χ*^*2*^ = 8.528, *df* = 1, *p* = 0.003) and M2 (*χ*^*2*^ = 4.407, *df* = 1, *p* = 0.036) (Figure S7).

**Figure 8:**
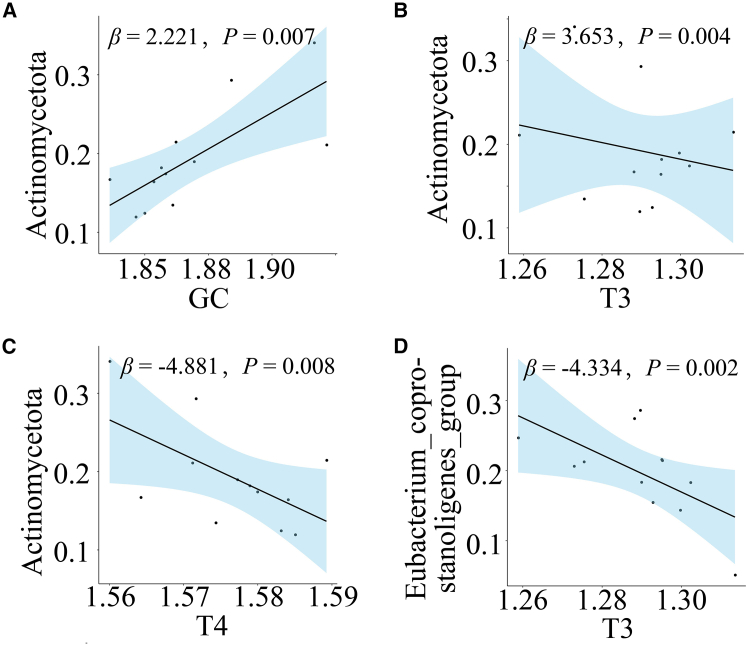
The correlations between hormones and the gut microbiome based on the GLM in different alpha male periods Only results with *p* ≤ 0.01 are presented here. (A) Correlation between GC and Actinomycetota. (B) Correlation between T3 and Actinomycetota. (C) Correlation between T4 and Actinomycetota. (D) Correlation between T3 and Eubacterium_coprostanoligenes_group. The Y-axis represents the relative abundance of microbial taxa (arcsine square-root transformation), and the X-axis represents hormone levels (log_10_(x) transformation).

GLM results further identified significant correlations between hormone levels and gut microbiome characteristics. At the phylum level, the proportion of Bacillota showed a significant negative correlation with T3 (*β =* −10.956, *p =* 0.036) (Figure S8A); the proportion of Actinomycetota was significantly positively correlated with GC (*β =* 2.221, *p =* 0.007) (Figure 8A), T3 (*β =* 3.653, *p =* 0.004) (Figure 8B), and significantly negatively correlated with T4 (*β =* −4.881, *p =* 0.008) (Figure 8C). At the family level, the proportion of Eubacterium_coprostanoligenes_group was significantly positively correlated with cortisol (*β =* 3.364, *p =* 0.022) (Figure S8F) and negatively correlated with T3 (*β =* −4.334, *p =* 0.002) (Figure 8D).

Functionally, the proportion of Organismal Systems exhibited a significant positive association with T3 (Level 1, *β =* 0.524, *p =* 0.047) (Figure S9A). The relative abundance of ko01200 (Carbon metabolism) is significantly negatively correlated with T4 (Level 3, *β* = −0.219, *p* = 0.042) (Figure S9B). In addition, the GC level was positively associated with Glycosyl Transferases (*β* = 1.417, *p* = 0.034) (Figure S9C) and GT4 (*β* = 1.009, *p* = 0.042) (Figure S9D); whereas GH109 was significantly negatively correlated with T4 (*β* = −1.414, *p* = 0.042) (Figure S9E); GT2_Glyco_tranf_2_3 showed significant positive correlations with GC (*β* = 0.314, *p* = 0.033) (Figure S9F) and cortisol (*β* = 0.531, *p* = 0.043) (Figure S9G). Other correlations between microbial taxa or functional genes and hormones based on GLM were presented in Table S10.

## Discussion

This study aims to investigate the potential associations between social environment changes and the gut microbiome of white-headed langurs in the study groups. Our results indicated that the structure and functional genes of the gut microbiome in these langurs exhibited significant differences across distinct social environments, which were also detected in the common marmosets (*Callithrix jacchus*),^34^ white-thighed colobus monkeys (*Colobus vellerosus*),^11^ and Verreaux’s sifakas (*Propithecus verreauxi*).^9^ Furthermore, some differences observed in this study may be associated with varying hormone levels between study groups, providing preliminary evidence of a potential link between social stress and gut microbiome.

### The comparison of gut microbiome of the mixed-sex group and the all-male group

Significant differences existed in the gut microbiome between mixed-sex and all-male groups. The all-male group exhibited stronger cellulose degradation potential in terms of structure and function, manifested as a compositional enrichment of more Bacillota, UCG-010, and Eubacterium_coprostanoligenes_group, and a functional enrichment of more pathways such as glycan biosynthesis and metabolism, ko00520, ko00010, as well as GT2_Glycos_transf_2 related to deglycosylation and glycosidic bond phosphorolysis. This observation could be related to the all-male group consuming more mature leaves, supporting prediction 1. A previous study indicated that the proportion of mature leaves consumed by the all-male group was significantly higher than that of mixed-sex groups.^29^ The Bacillota is highly associated with the digestion of cellulose.^32^^,^^35^ A study in mice has found that the abundance of Eubacterium_coprostanoligenes_group is positively correlated with the content of butyrate in feces.^36^ The relative abundance of UCG-010 in the gut of snowshoe hares (*Lepus americanus*) increases while fecal fiber content correspondingly decreases.^37^ These bacterial taxa possess the potential to digest cellulose and produce SCFAs, adapting to the higher proportion of mature leaves consumed by the all-male group. Research on Tibetan macaques *(M. mulatta vestita*) has revealed significant enrichment of glycolysis/gluconeogenesis pathways in their gut microbiome, which may be related to improved energy production efficiency as an adaptive response to the consumption of lower-quality food in high-altitude habitats.^38^ Similarly, the enrichment of functional genes related to polysaccharide metabolism in all-male groups may also be a response to their high intake of low-quality food.

Furthermore, the stronger ability of the gut microbiome to digest plant polysaccharides may be linked to the higher emergency stress and energy demand in all-male groups. The results of GLM indicated that an increase in cortisol and T3 levels were associated with increased relative abundances of Bacillota, Eubacterium_coprostanoligenes_group, and Ruminococcaceae. Functionally, the biosynthesis and metabolism of glycans, as well as the gene content of the ko00520 pathway, were also significantly positively correlated with T3 level, supporting prediction 2. Increased GC secretion rapidly mobilizes the organism’s energy reserves to cope with stress, while elevated thyroid hormone levels raise the basal metabolic rate to meet its energy demands.^39^^,^^40^ A study of chimpanzees (*Pan troglodytes*) has indicated that aggressive individuals expend more energy and experience significantly increased stress levels.^41^ In our study, the hormone secretion levels in the all-male group were significantly higher than those in the mixed-sex group. Faced with pressures such as competing for reproductive rights and being chased by the mixed-sex group, the all-male group may secrete hormones to mobilize energy for stress responses or to execute aggressive behaviors.^41^ The enrichment of cellulose-digesting bacteria and metabolic pathways related to polysaccharide degradation may facilitate the degradation of complex plant polysaccharides and SCFAs production, potentially supporting host energy supply.^32^^,^^36^^,^^42^

Compared to the all-male group, the mixed-sex group exhibited significantly higher alpha diversity indices, while there was no difference in richness was observed, indicating that the relative abundance of the gut microbiome in the mixed-sex group was more uniform. This could be attributed to two potential reasons. Firstly, as the permanent members of the mixed-sex group consist of females with kinship ties, vertical bacterial transmission to offspring likely leads to higher similarity in gut microbiome composition among group individuals.^43^ Secondly, previous studies have illustrated that the mixed-sex groups have smaller home ranges than all-male groups and typically larger group sizes.^29^ Consequently, the mixed-sex group may have more opportunities for horizontal transmission within shared environments, thereby enhancing gut microbiome uniformity.^13^^,^^29^

Moreover, the gut microbiome of the mixed-sex group showed enrichment in Actinomycetota and Pseudomonadota. In potential function, they exhibited higher gene proportions of KEGG pathways, such as energy metabolism, other amino acid metabolism, and metabolism of terpenoids and polyketides (Level 2); ko01100 and ko01200 (Level 3), and CE10 enzyme genes acting on non-carbohydrates.^44^^,^^45^ These results may be related to mixed-sex groups consuming more young leaves and the reproductive demands of females, providing further evidence supporting prediction 1. Previous studies have demonstrated that mixed-sex groups consume more young leaves rich in soluble carbohydrates and proteins than all-male groups.^29^^,^^46^ The Pseudomonadota contribute to the digestion and metabolism of fats and proteins.^47^ And the Actinomycetota degrade mucins and enhance intestinal mucosal permeability to promote nutrient absorption.^48^ Studies have found that the abundance of these two taxa gradually increases during pregnancy, supporting fetal growth and maternal lactation.^47^^,^^49^^,^^50^ Meanwhile, metabolites of amino acids could regulate the brain-gut axis, facilitate maternal pregnancy adaptation, and promote placental formation and fetal development.^51^^,^^52^ Therefore, the enrichment of Pseudomonadota, Actinomycetota, and functional genes related to amino acid metabolism in mixed-sex groups may contribute to enhanced digestion and absorption of proteins and carbohydrates in young leaves, potentially providing energy for reproductive activities. This digestive strategy mirrors that of wild foraging groups of Yunnan snub-nosed monkeys (*Rhinopithecus bieti*),^53^ addressing the high energy demands of low-quality habitats by enhancing the metabolic capacity for non-carbohydrate nutrients.

Notably, we found that prolonged high-stress states may be accompanied by changes in gut microbiome function genes. First, the relative abundance of the Pseudomonadota and its potentially pathogenic family, the Moraxellaceae, showed a significant positive correlation with T4 level. Second, the genes’ relative abundances of Cellular Processes, cellular community - prokaryotes, drug resistance: antimicrobial, and nervous system, were significantly negatively correlated with cortisol, T3, and T4 levels. Conversely, the gut microbiome may also influence host hormone levels through microbial metabolism and immune activation, forming a bidirectional axis between microbes and the endocrine system.^23^ Indeed, the gut microbiome interacts with the host’s neural and endocrine systems through the production of metabolites, thereby modulating host growth, development, and metabolism.^23^^,^^54^^,^^55^^,^^56^ This is similar to the findings in Asian elephants (*Elephas maximus*), where elevated thyroid hormone levels promote increased relative abundance of most potential pathogens, such as *Treponema* sp.^57^ However, the present short-term data do not allow us to infer causal or directional links between hormones and the gut microbiome. Future longitudinal monitoring of gut microbial dynamics, hormone levels, and their relationships is thus encouraged.

### The comparison of the gut microbiome of the different male periods

Our results indicated that the composition of the gut microbiome of white-headed langurs varies across different alpha male periods, supporting prediction 3. First, the relative abundance of the Actinomycetota was significantly higher in M1 than in M2 and M3, potentially related to the langurs' frequent foraging in cultivated fields and the highest GC levels observed during the M1 period. Studies on yellow baboons (*Papio cynocephalus*) and anubis baboons (*P. Anubis*) have revealed that soil microorganisms shape the structure of the primate gut microbiome.^58^ As a major group of soil microorganisms, the Actinomycetota could be promoted to colonize the host’s gut through the intake of food near the soil surface or the mutual licking of hair contaminated with soil.^58^ In our observations, the study group frequently foraged in sugarcane fields for herbaceous plants such as *Youngia japonica*, providing a pathway for the spread of Actinomycetota into their gut. Additionally, the GLM results showed that the proportion of Actinomycetota was significantly positively correlated with GC. Previous studies have suggested that elevated GC secretion may be associated with intestinal mucosal permeability and could potentially facilitate bacterial translocation across the gut barrier.^59^^,^^60^^,^^61^ During the M1, the study group may have undergone frequent intrusions by outside males, which could induce physiological stress and subsequently activate the HPA axis, leading to relatively higher GC level. Consequently, the changes in intestinal permeability might be linked to the increased colonization of soil-derived Actinomycetota.

M2 was characterized by a high abundance of Eubacterium_coprostanoligenes_group, with its relative abundance significantly positively correlated with cortisol level. This finding could be related to infant langur disappearances, supporting prediction 4. In this study, the cortisol level was the highest during M2, which may be associated with infanticide events. Our observations revealed that during M2, the new alpha male frequently exhibited behaviors of harming infant langurs, and instances of infant disappearance were also recorded. Studies on chacma baboons (*Papio hamadryas ursinus*)^62^ and white-faced capuchins (*Cebus capucinus*)^63^ have shown that increased infanticide risk leads to elevated stress hormone secretion in female primates. In particular, cortisol participates in regulating various physiological activities, including glucose and lipid metabolism, mobilizing energy to complete stress responses.^64^ Previous evidence has indicated that the Eubacterium_coprostanoligenes_group is associated with butyrate production.^36^ Thus, the enrichment of this taxon in M2 may reflect enhanced utilization of nutritional substrates for butyrate generation, potentially supporting host energy supply in response to stress and energy deficits associated with infanticide events.

The abundance of the Bacteroidota was significantly the highest in M3, which may be related to the higher food quality during this period. Bacteroidota, closely involved in carbohydrate and protein metabolism, aid hosts in catabolizing simple carbohydrates when high-quality food becomes more accessible seasonally.^65^^,^^66^ White-headed langurs increase their consumption of young leaves and fruits rich in simple carbohydrates during the rainy seasons.^67^^,^^68^ In our study, rainfall peaked during the M3. As rainfall increased, the availability of high-quality food in the limestone forest rose, potentially resulting in higher simple carbohydrate intake by the langurs.^67^^,^^68^ Additionally, we observed changes in the home range size of the study group. Their frequent foraging on young leaves of cultivated plants such as *Hibiscus rosa-sinensis*, and *Zanthoxylum armatum* during this period, likely provided these langurs with more high-quality carbohydrates, promoting the growth of the Bacteroidota. Moreover, the community richness index of gut microbiome in M3 was significantly higher than in the preceding two periods, potentially linked to increased rainfall and enlarged home ranges enhancing food diversity.

In terms of potential function, gut microbiome in M1 and M2 showed similarity, while M3 exhibited significant differences from the earlier periods. This is primarily manifested in the significantly lower proportion of Metabolism genes in M3 compared to M1 and M2, including genes of Level 2 pathways such as global and overview maps, energy metabolism, other amino acid metabolism, and Level 3 genes such as ko01100 and ko01200. These findings indicate a lower metabolic potential in the gut microbiome of the langurs in M3, which potentially relates to improved food quality and physiological regulatory changes. By altering rainfall patterns and home range changes during M3, environmental conditions might provide these langurs greater access to high-quality carbohydrates (e.g., starch and fat), substrates that are directly digestible and utilized by host enzymes for energy.^67^^,^^68^^,^^69^ For instance, starch is digested by salivary amylase and pancreatic amylase into maltose and isomaltose, which are subsequently hydrolyzed into glucose for cellular respiration and energy supply.^69^ Therefore, greater intake of high-quality dietary nutrients may be linked to reduced metabolic potential of the gut microbiome. Additionally, the relative abundance of multiple enzyme genes associated with complex plant polysaccharide metabolism, such as GH3, GH78, and AA3, was significantly lower in M3 than in M1 and M2, further supporting the aforementioned findings.

Furthermore, we found that Human Disease genes were more highly enriched in M3, which might be related to the fact that the study groups changed the sleeping site and conducted foraging in the reserve. Human Disease genes could be transferred to the host through environmental microorganisms.^70^ During M3, the study group relocated their sleeping site to mountainous areas adjacent to the reserve and frequently exhibited foraging patterns involving traversing farmland to enter the reserve. Natural potential pathogens in farmland soils, combined with those from agricultural activity residues, significantly increase the risk of pathogen exposure during foraging by the langurs.^71^ Besides, the frequent foraging of the langurs within the reserve increases their likelihood of encountering visitors.^71^ Close human-langur interactions may elevate the risk of bidirectional transmission of zoonotic diseases, thereby contributing to an enrichment of Human Disease genes in the gut microbiome of the langurs.^71^

### Limitations of the study

Our study indicated that gut microbiome differences under diverse social environments could be the result of the combined effects of multiple factors, including hormone levels, dietary composition, and reproductive demands, providing valuable socioecological insights for the conservation of white-headed langurs. However, we acknowledged that there are limitations in the study. First, due to the species' inaccessible cliff-dwelling behavior and sensitivity to human disturbance, we were unable to implement effective individual identification methods. Consequently, age and sex could not be controlled in our analyses, which might introduce potential biases, particularly for comparisons involving mixed-sex groups. Moreover, because fecal samples could not be matched to specific individuals, the hormone–microbiome association analyses in this study can only reflect statistical correlations at the group level. This limitation resulted in the loss of key sociological data, such as social network structures, thereby hindering an in-depth analysis of the complex mechanisms linking social factors to the gut microbiome. While we acknowledge this as a major limitation of the present study, future research should aim to overcome the bottleneck of individual identification to systematically collect age, sex, and social interaction data among group members. This would enable the construction of social networks and allow a deeper understanding of how social factors influence the gut microbiome. Second, this study only preliminarily explored differences in the gut microbiome using a limited number of groups, and the generalizability of our findings is restricted. Therefore, this study can only provide preliminary evidence of a correlation between the social environment (including different social group types or alpha male replacement) and fluctuations in the gut microbiome, and we are unable to completely rule out the effects of other group-specific events on the results. Independent validation across more study groups and the establishment of a longitudinal sampling framework are therefore needed in future work. In addition, given the profound influence of diet on the gut microbiome, this study did not systematically record dietary composition. Furthermore, some samples had already been depleted in the earlier sequencing stage. Therefore, we are unable to rule out that dietary differences, rather than social environment per se, drive the observed microbial differences. Future studies should collect fecal samples for metabarcoding or conduct feeding observations to disentangle these effects. Finally, the functional genes identified in this study suggest potential functions, which could be further explored using transcriptomic and metabolomic approaches.

Despite these limitations, our findings suggest that gut microbiome flexibility may help hosts adapt to social and dietary changes. For endangered species in fragmented habitats, microbial plasticity could serve as an adaptive mechanism to buffer nutritional stress. Monitoring the dynamics of the gut microbiome can provide complementary evidence for the conservation assessment of endangered species.

## Resource availability

### Lead contact

Further information and requests for resources and reagents should be directed to and will be fulfilled by the Lead Contact, Zhonghao Huang (hzh773@126.com).

### Materials availability

This study did not generate new unique reagents.

### Data and code availability

• The raw sequencing data during the current study are available in the NCBI repository at https://www.ncbi.nlm.nih.gov, and the SRA accession numbers are PRJNA1431103 (16S rRNA sequences) and PRJNA1431660 (metagenomic sequences).
• Code reported in this paper is available as Data S1.
• Any additional information required to reanalyze the data reported in this paper is available from the lead contact upon request.

## Acknowledgments

This study was supported by the substantial help given by Mr. Dengpan Nong, Mr. Shijun Wu, Mr. Jipeng Liang, and Mr. Zhizhang Liang from the Administration Center of Guangxi Chongzuo White-headed Langur National Nature Reserve. We thank Ms. Hongying Liu (from 10.13039/501100004194Northwest University, China), Ms. Fengxiang Mo and Ms. Yujing Qiu (from 10.13039/501100009007Guangxi Normal University, China) for their help in fecal sample collection and conservation. This work was supported by the 10.13039/501100001809National Natural Science Foundation of China (grant nos. 32560126, 32170488), the Guangxi Forestry Science and Technology Promotion Project, China (grant no. 2026GXLK34), the Guangxi Natural Science Foundation, China (grant no. 2026GXNSFHA00640091), and the Improvement of Basic Scientific Research Ability of Young and Middle-Aged Teachers in Guangxi Universities, China (grant no. 2023KY0559).

## Author contributions

Z.H. designed the study. Y.C. collected samples, analyzed the data, and wrote the manuscript. Y.L. collected samples and analyzed the data. Z.L., J.Z., K.Z., and S.L. collected samples and revised the manuscript. Z.H. and S.L. revised the manuscript. All authors read and approved the submitted manuscript.

## Declaration of interests

The authors declare no competing interests.

## STAR★Methods

### Key resources table

**Table:** 

REAGENT or RESOURCE	SOURCE	IDENTIFIER
**Critical commercial assays**		

E.Z.N.A.® soil DNA Kit	Omega Bio-tek	N/A
AxyPrep DNA Gel Extraction Kit	Axygen Biosciences	N/A
NEXTFLEX Rapid DNA-Seq Kit	Bioo Scientific	N/A
GC ELISA research kit	Jiangsu Jingmei Biotechnology Co., Ltd.	J87011-A
Cortisol ELISA research kit	Jiangsu Jingmei Biotechnology Co., Ltd.	J87021-A
T3 ELISA research kit	Jiangsu Jingmei Biotechnology Co., Ltd.	J87014-A
T4 ELISA research kit	Jiangsu Jingmei Biotechnology Co., Ltd.	J87016-A

**Deposited data**		

Raw sequencing data (16S rRNA sequences)	This paper, https://www.ncbi.nlm.nih.gov	PRJNA1431103
Raw sequencing data (metagenomic sequences)	This paper, https://www.ncbi.nlm.nih.gov	PRJNA1431660
Analyzed data	This paper	Supplementary tables

**Software and algorithms**		

Fastp v 0.19.6, v0.20.0	Magoč et al.^1^	https://github.com/OpenGene/fastp
FLASH v 1.2.11	Edgar, R.^2^	http://www.cbcb.umd.edu/software/flash
mothur	Schloss Patrick et al.^3^	http://www.mothur.org/wiki/Calculators
Megahit v 1.1.2, v1.2.9	Li et al.^4^	https://github.com/voutcn/megahit
Prodigal v2.6.3	Hyatt et al.^5^	https://github.com/hyattpd/Prodigal
CD-HIT v 4.7	Fu et al.^6^	http://weizhongli-lab.org/cd-hit/
Diamond v2.0.13	Buchfink et al.^7^	https://github.com/bbuchfink/diamond
SOAPaligner v soap2.21release	Li et al.^8^	https://github.com/ShujiaHuang/SOAPaligner

**Other**		

T100 Thermal Cycler PCR thermocycler	BIO-RAD	N/A
Qubit 4.0	Thermo Fisher Scientific	N/A
Miseq PE300 platform	Illumina	*N/A*
Illumina HiSeq platform	Illumina	N/A
Sliva 16S rRNA gene database	N/A	https://www.arb-silva.de/
KEGG database	N/A	https://www.genome.jp/kegg
CAZy database	N/A	http://bcb.unl.edu/dbCAN2/download/Datab

### Experimental model and study participant details

This study was conducted in Guangxi Chongzuo White-headed Langur National Nature Reserve, China. The annual average temperature at the research site is 21.8°C, and the total annual rainfall is 1370.0 mm (Figure S10). The year can be divided into four seasons (spring: March to May, summer: June to August, autumn: September to November, winter: December to February). The subjects were the four groups of whited-headed langurs in Banli area of this reserve, including three mixed-sex groups (SKS, NS, and ZWY) and one all-male group (HS). Among these groups, SKS and NS represented the mixed-sex groups, whose gut microbiome were compared with the all-male group (HS). The sampling period was from April 2021 to November 2021. During this period, SKS and NS had 14 individuals respectively, and HS had 13 individuals. Additionally, ZWY experienced two alpha male replacements (in June 2023 and July 2023) from March 2023 to February 2024. We collected feces from the ZWY groups to investigate the impact of the alpha male replacements on the gut microbiome, and divided the study period into three periods based on the tenure of the alpha males: M1 (38 individuals), M2 (25-27 individuals), and M3 (21-23 individuals).

This study was performed in accordance with the guidelines of Institutional Animal Care and Use Committee (IACUC) and was approved by the Laboratory Animal Care and Animal Ethics Committee of Guangxi Normal University (permit No.202103-032). All fecal samples were collected with permission from the Administration Center of Guangxi Chongzuo White-headed Langur National Nature Reserve. We did not collect any animal tissue samples that might cause injury or distress to the langurs. Instead, fecal samples were collected after the langurs left their sleeping sites to avoid inducing any stress responses.

### Method details

#### Sample collection information

The white-headed langur groups defecate before leaving their sleeping sites in the early morning. We arrived at the base of the cliff after the langurs had left to collect fresh fecal samples. The samplers wore sterile gloves and masks throughout the sampling process. When sampling, fresh and intact feces were selected, and the parts that had not been in contact with the external environment were picked up with sterile bamboo sticks. Each sample was taken in 3-5 g and placed in a sterile collection tube labeled with sampling information. The samples were transferred to a -80°C ultra-low temperature refrigerator using a dry ice box for preservation. During sampling, each sample was spaced at least 2 m to minimize the possibility of collecting feces from the same individual. The frozen samples were transported to the laboratory in sufficient dry ice for molecular biological experiments.

Our study collected a total of 334 fecal samples, including 186 samples to compare the gut microbiome of mixed-sex group and the all-male group of the white-headed langurs (the mixed-sex group:126 samples, including 70 samples from NS, 56 samples from SKS; the all-male group: 60 samples). Additionally, the 148 samples of ZWY to compare the differences in gut microbiome in different alpha male periods (M1: 43 samples; M2: 25 samples; M3: 80 samples) (Table S11).

Collecting hormone fecal samples simultaneously with those of the gut microbiome. A total of 121 samples were used for comparison between the mixed-sex group and the all-male group, including 84 samples from the mixed-sex group and 37 samples from the all-male group (Table S11). In addition, we collected 116 hormone samples for comparison among different alpha male periods, including 33 samples from M1, 21 samples from M2, and 62 samples from M3 (Table S11).

#### 16S rRNA sequencing and metagenomic sequencing

Sample DNA was extracted using the E.Z.N.A.® Soil DNA Kit. DNA quality was assessed via 1% agarose gel electrophoresis, and purity and concentration were analyzed using a Thermo NanoDrop 2000 micro-volume UV spectrophotometer. The V3–V4 region of bacterial 16S rRNA was amplified via Polymerase Chain Reaction (PCR), using the primers (338F: 5’-ACTCCTACGGGAGGCAGCAG-3’; 806R: 5’-GGACTACHVGGGTWTCTAAT-3’). The PCR system contained 5×FastPfu Buffer (4 μL), 2.5 mM dNTPs (2 μL), 5 μM forward primer (0.8 μL), 5 μM reverse primer (0.8 μL), FastPfu Polymerase (0.4 μL), BSA (0.2 μL), template DNA (10 ng), and ddH_2_O (added to a final volume of 20 μL).The PCR amplification was performed in three stages: initial denaturation (95°C for 3 min); denaturation and annealing (28 cycles of 95°C for 30 s, 53°C for 30 s, and 72°C for 45 s); final extension and hold (72°C for 10 min, then held at 10°C for 10 min. After three amplifications for each sample, the products were mixed and subjected to 2% agarose gel electrophoresis to obtain the PCR products. Then, the products were purified and quantified using the AxyPrep DNA Gel Extraction Kit and Quanti Fluor™-ST (Promega Company). The fluorescence-quantified products were libraryed using the NEXTFLEX Rapid DNA-Seq Kit and sequenced on the Miseq PE300 platform (Illumina Company).

Quality control of raw sequences was performed using Fastp (https://github.com/OpenGene/fastp,v 0.19.6) software.^72^ Splicing of sequences was conducted in Flash (http://www.cbcb.umd.edu/software/flash,v 1.2.11) software.^73^ The DADA2 plugin in the Qiime2 (https://qiime2.org) workflow is used to perform noise reduction on the optimized sequences, removing erroneous or repetitive sequences to obtain the Amplicon Sequence Variant (ASV). The ASV sequences were then aligned to the Sliva 16S rRNA gene database (https://www.arb-silva.de/) to obtain the microbial classification information of the samples. Finally, secondary drawdown based on the minimum sequence number was performed for subsequent analysis.

Based on 16S rRNA sequencing results, three representative fecal samples with the highest alpha diversity were selected from each group per month for metagenomic sequencing. A total of 78 samples were used for metagenomic sequencing. First, the DNA in the samples was extracted and subjected to 1% agarose gel electrophoresis to detect its quality. Then, the quality-checked DNA was fragmented using a Covaris M220 ultrasonic disintegrator. The resulting 400 bp gene fragments underwent adapter ligation, magnetic bead selection, PCR amplification, and magnetic bead recovery before library preparation using the NEXTFLEX™ Rapid DNA-Seq Kit. Finally, DNA from the library underwent bridge PCR amplification. Sequences of the template DNA fragments were obtained using four fluorescently labeled dNTPs and sequenced on the Illumina HiSeq platform.

Using the Fastp (https://github.com/OpenGene/fastp, v 0.20.0) software,^72^ primer fragments were trimmed to retain high-quality sequence fragments longer than 50 bp, with an average quality score above 20 and no N bases. After removing host sequences, the sequences were assembled using the Megahit (https://github.com/voutcn/megahit, v 1.1.2) software.^74^ Open reading frames (ORFs) were predicted within the contigs from the assembly results using the Prodigal (https://github.com/hyattpd/Prodigal,v 2.6.3) software.^75^ Genes with nucleotide lengths ≥100 bp were selected and translated into amino acid sequences to obtain gene prediction results for each sample.

All the gene sequences of the samples were clustered using the CD-HIT (http://weizhongli-lab.org/cd-hit/,v 4.7) software.^76^ The longest gene in each cluster was selected as the representative sequence to construct a non-redundant gene set. Each sample's high-quality reads were compared with the non-redundant gene set using the SOAPaligner (https://github.com/ShujiaHuang/SOAPaligner,v soap2.21release) software,^77^ and the abundance information of genes in the corresponding samples was calculated. The non-redundant gene set was compared to the KEGG (Kyoto Encyclopedia of Genes and Genomes,https://www.genome.jp/kegg) database and CAZy (Carbohydrate-Active enZYmes Database,http://bcb.unl.edu/dbCAN2/download/Datab) database using the Diamond (https://github.com/bbuchfink/diamond,v 2.0.13, an e-value cutoff of 1e-5) software to obtain the abundance of functional genes.

#### Hormone measurement

The Enzyme-linked Immunosorbent Assays (ELISAs) were applied to measure GC, cortisol, T3, and T4 levels in fecal samples (Table S11). A solid-phase antibody is prepared by coating a microplate with purified antibodies for the analyte hormone. The analyte hormone is added successively to the wells coated with the monoclonal antibody, and then it combines with the corresponding antibody labeled with Horseradish Peroxidase (HRP) to form an antibody-antigen-enzyme-labeled antibody complex. After thorough washing, the substrate 3,3',5,5'-Tetramethylbenzidine (TMB) is added. This substance can be converted into blue under the catalysis of HRP enzyme and then into yellow under the action of acid. The depth of the color is positively correlated with the content of the analyte hormone in the sample. The absorbance (OD value) is measured using an enzyme detector at a wavelength of 450 nm. The final concentration of hormone can be calculated using the standard curve. Differences in the four hormones across social groups were assessed using the GLMM. Season was treated as a random factor, different communities as fixed effects, and hormone levels as response variables.

### Quantification and statistical analysis

#### Comparative analysis of gut microbiome

Generalized Linear Mixed Models (GLMM) were constructed to compare differences in the gut microbiome of white-headed langurs across distinct social environments (mixed-sex group vs. all-male group, different alpha male periods). Given that season is a key environmental variable influencing primate diet and physiology, we included it as a random effect to control for its potential impact.^31^ We used the different social groups as fixed effects. Response variables comprised diversity indices, relative abundances of bacterial taxa and functional genes. Analysis of variance (ANOVA) was used to determine whether models with or without fixed effects showed significant differences, thereby assessing whether response variables varied significantly across fixed-effect groupings. In addition, we compared the differences in the gut microbiome in the white-headed langurs across three periods (M1, M2, M3) using the Kruskal-Wallis test (K-W test), with the *p*-values subjected to multiple correction via the false discovery rate (FDR) method. The *p* < 0.05 indicates that the response variables varied significantly across the defined fixed-effect categories (∗ for *p* < 0.05, ∗∗ for *p* < 0.01, and ∗∗∗ for *p* < 0.001).

Principal Coordinates Analysis (PCoA) based on the Bray-Curtis distance algorithm was used to examine differences in the gut microbiome structure and functional genes under different social environments. The contribution of grouping factors to sample variations and their significance levels were assessed by Permutational MANOVA (R, v 3.3.1 and v 4.4.0; a significance level of *p* < 0.05: ∗ for *p* < 0.05, ∗∗ for *p* < 0.01, and ∗∗∗ for *p* < 0.001). Additionally, a Random Forest (RF) model with 500 decision trees was constructed to identify representative bacterial taxa distinguishing gut microbiome between social groups at the phylum and family levels. The RF classification performance was evaluated using the pROC package to compute Receiver Operating Characteristic (ROC) curves and Area Under the Curve (AUC) values, where AUC values closer to 1 indicated stronger predictive capability.

#### Correlation between hormones and gut microbiome

We conducted the Generalized Linear Model (GLM) to assess the correlation between hormones and the relative abundances of dominant bacterial taxa and functional genes (> 1%). Before this, Spearman's correlation analysis was performed to examine the correlations among the hormones, given that some of the data deviated from a normal distribution. The results indicated the absence of strong correlations between the hormones (|r| < 0.80). In the GLM, the levels of the four hormones served as explanatory variables, and alpha diversity, relative abundance of major bacterial taxa and functional genes were set as response variables. The *calc.relimp* function (relaimpo package, R v 4.4.0) was used to quantify the relative importance of explanatory variable.

Data for GLMM and GLM in the STAR Methods section were transformed to improve linearity: relative abundance were arcsine square-root transformation, while diversity indices, hormone levels and other data were log_10_(x) transformation. GLMM was built with the *lmer* function (lme4 package), and GLM with the *glm* function (R base package). All models used a significance level of *p* < 0.05 (∗ for *p* < 0.05, ∗∗ for *p* < 0.01, and ∗∗∗ for *p* < 0.001).

## Supplemental information

Document S1. Figures S1–S10

Table S1. The information of 16S rRNA and metagenomic sequencing of all samples in the white-headed langursʹ gut microbiome (M1: the period dominated by alpha male 1, M2: the period dominated by alpha male 2, M3: the period dominated by alpha male 3)

Table S2. The relative abundance of composition and functional genes of the gut microbiome in white-headed langurs

Table S3. The comparison of gut microbiome structure between mixed-sex group and all-male group

Table S4. The comparison of KEGG pathways of the gut microbiome between the mixed-sex group and the all-male group

Table S5. The comparison of CAZy genes of the gut microbiome between mixed-sex group and all-male group

Table S6. The correlations between hormones and the gut microbiome structure, functional genes in the mixed-sex group and all-male groups based on the GLM

Table S7. The comparison of gut microbiome structure in different alpha male periods (M1: the period dominated by alpha male 1, M2: the period dominated by alpha male 2, M3: the period dominated by alpha male 3)

Table S8. The comparison of KEGG pathways of the gut microbiome in different alpha male periods (M1: the period dominated by alpha male 1, M2: the period dominated by alpha male 2, M3: the period dominated by alpha male 3)

Table S9. The comparison of CAZy genes of the gut microbiome in different alpha male periods (M1: the period dominated by alpha male 1, M2: the period dominated by alpha male 2, M3: the period dominated by alpha male 3)

Table S10. The correlations between hormones and the gut microbiome structure, functional genes in different alpha male periods based on the GLM

Table S11. Sampling information for study groups of the white-headed langurs (M1: the period dominated by alpha male 1, M2: the period dominated by alpha male 2, M3: the period dominated by alpha male 3)

Data S1. Main code and related information
