# Supplementary material for: The adaptation of the gut microbiome to social environmental changes in an Asian langur
Source: iScience. 2026 Jul 21;29(8):116779. doi: 10.1016/j.isci.2026.116779 (PMC13392869; doi:10.1016/j.isci.2026.116779)
Supplement: Document S1. Figures S1–S10 [file mmc1.pdf]

iScience, Volume *29*

Supplemental information

The adaptation of the gut microbiome to social environmental changes in an Asian langur

Yanqiong Chen, Ying Lai, Zheng Liu, Kechu Zhang, Jingjin Zheng, Shiyi Lu, and Zhonghao Huang

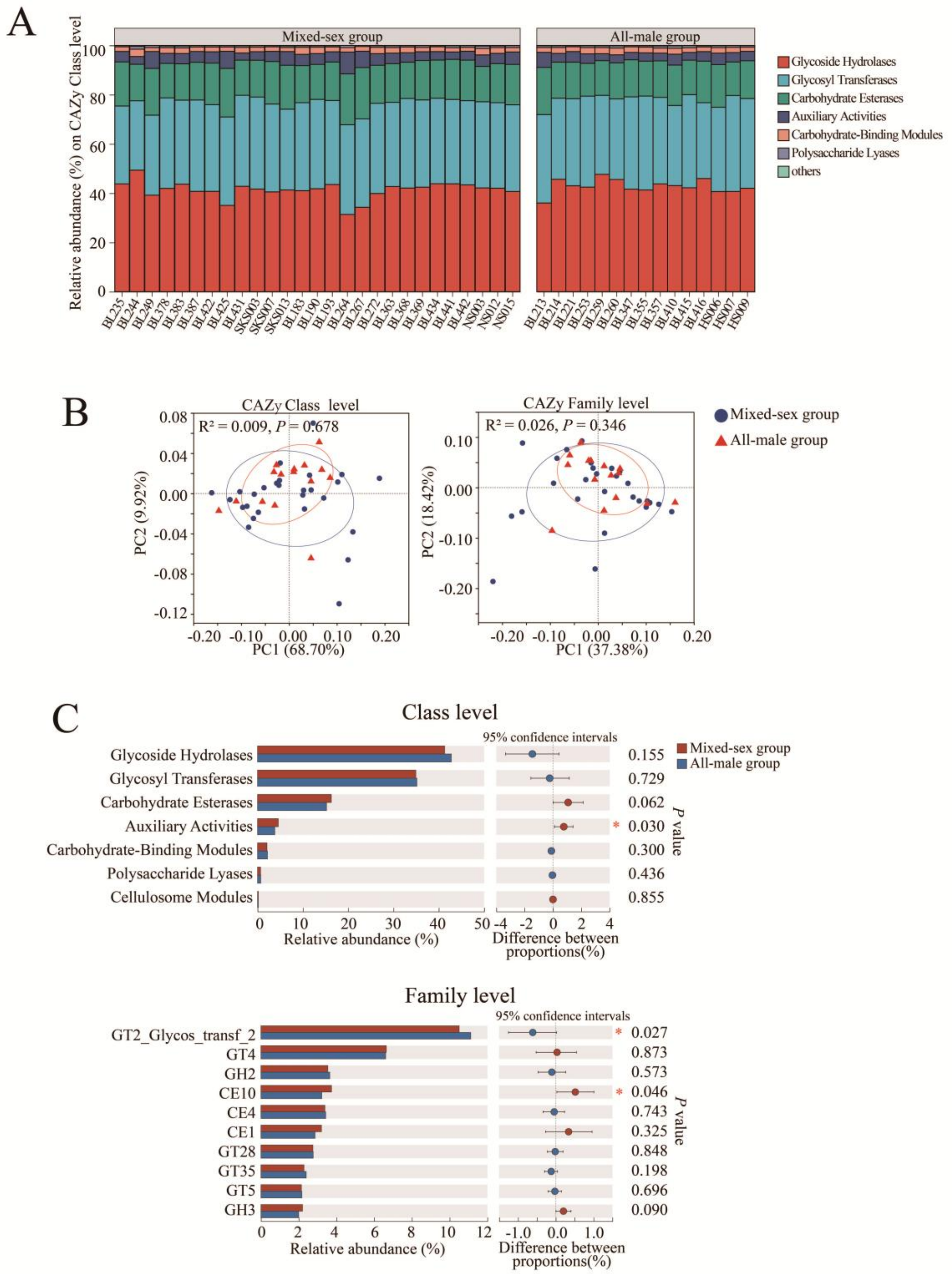

Figure S1. The CAZy genes composition of gut microbiome in mixed-sex group and all-male group (a) ("others" includes all taxa whose relative abundance is less than 1%), the PCoA results in Class and Family (b) levels, and comparison of CAZy genes based on GLMM (c) (* for $P < 0.05$, ** for $P < 0.01$, and *** for $P < 0.001$)

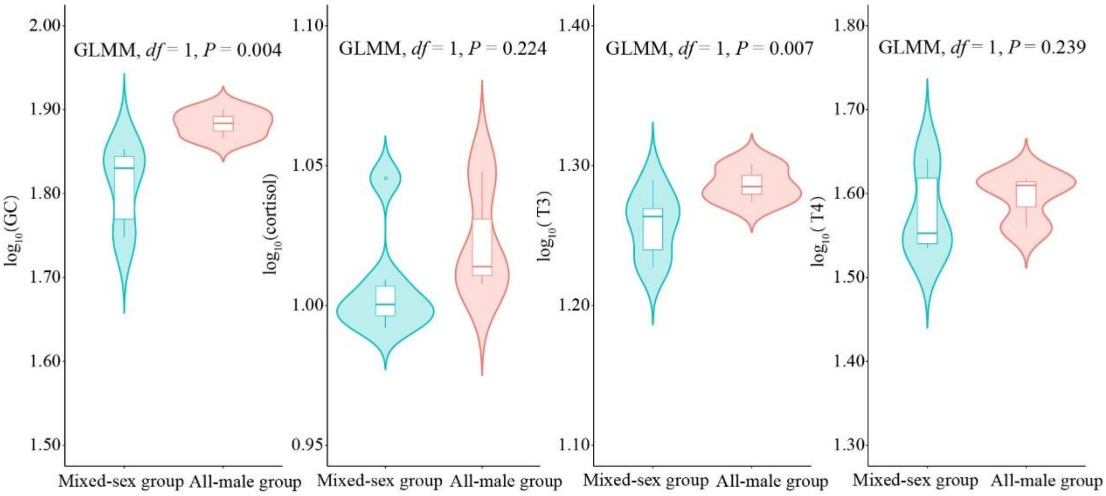

Figure S2. The comparison of hormones between mixed-sex group and all-male group based on GLMM

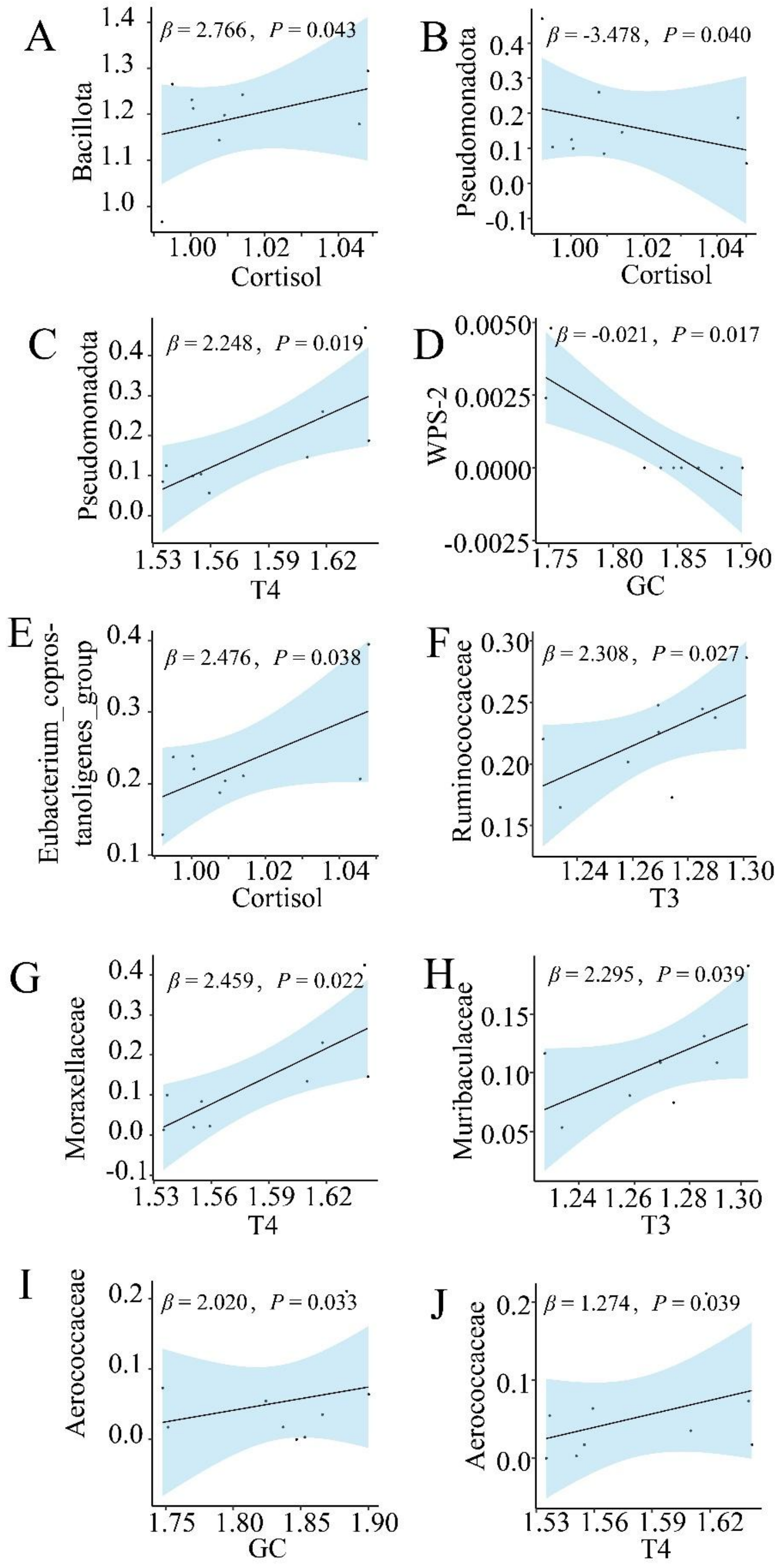

Figure S3. The correlations between hormones and the gut microbiome structure in mixed-sex and all-male groups based on the GLM (only results with $0.01 < P \leq 0.05$ are presented here). The Y-axis represents the relative abundance of microbial taxa (arcsine square-root transformation), and the X-axis represents hormone levels ($\log_{10}(x)$ transformation)

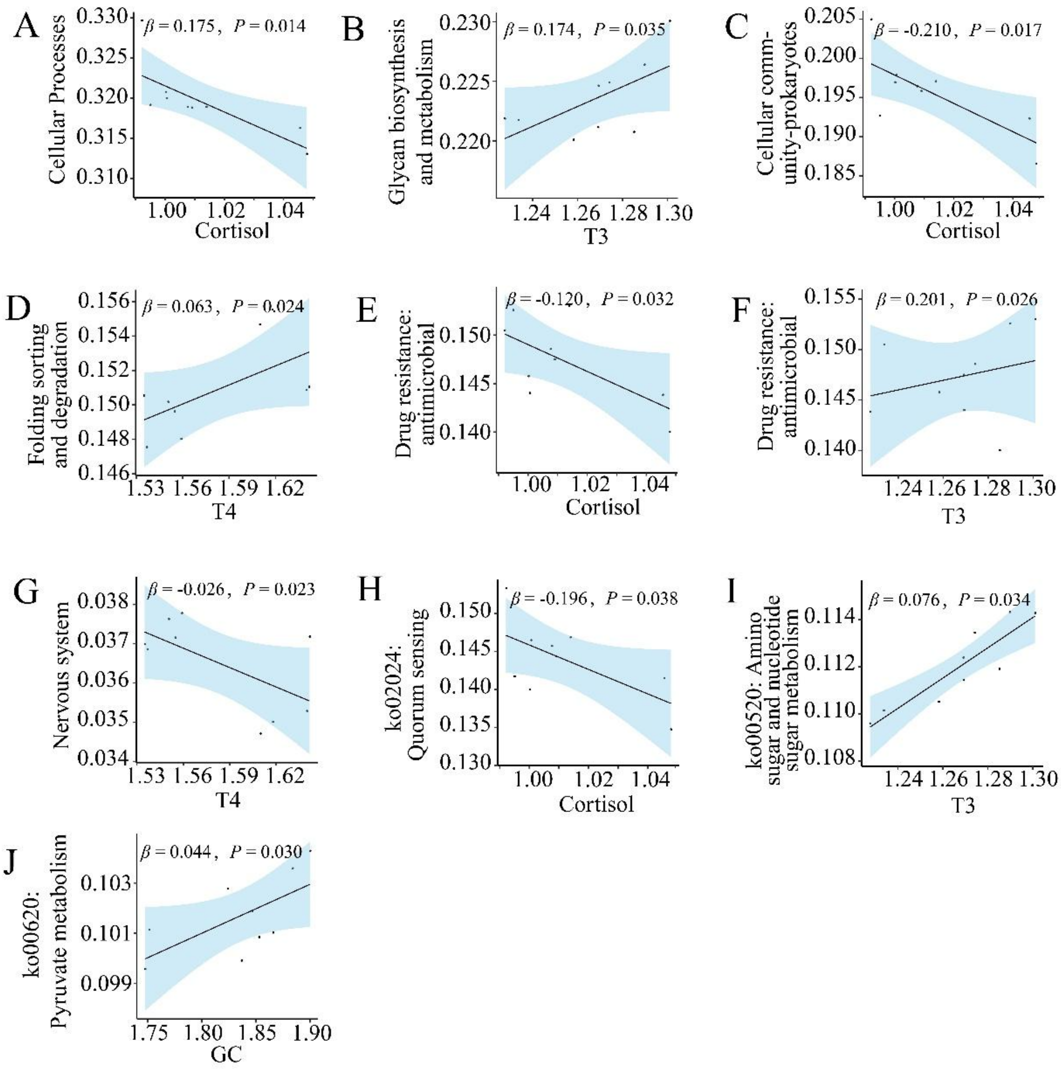

Figure S4. The correlations between hormones and the gut microbiome KEGG functional genes in mixed-sex and all-male groups based on the GLM (only results with $0.01 < P \leq 0.05$ are presented here). The Y-axis represents the relative abundance of microbial taxa (arcsine square-root transformation), and the X-axis represents hormone levels ($\log_{10}(x)$ transformation)

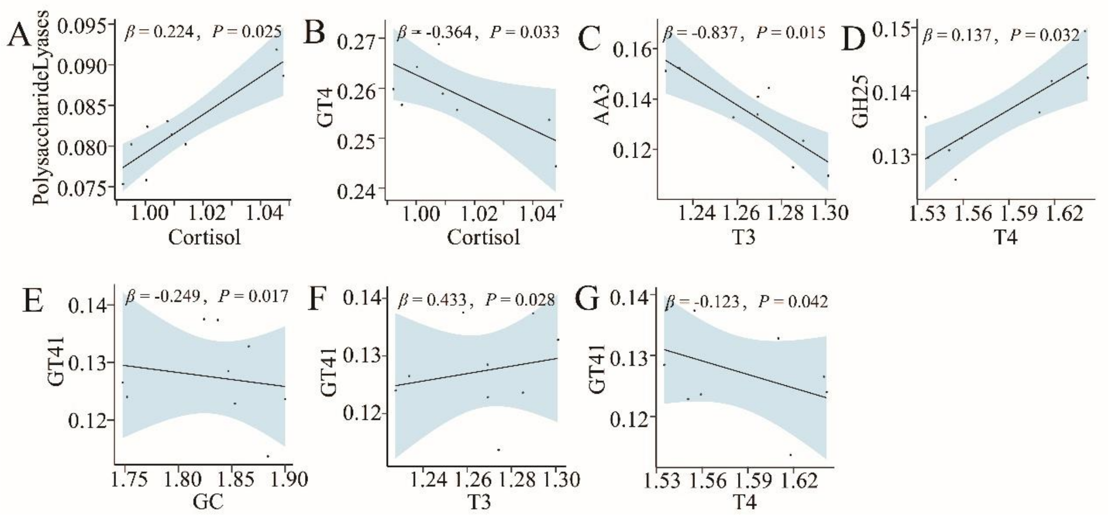

Figure S5. The correlations between hormones and the gut microbiome CAZy functional genes in mixed-sex and all-male groups based on the GLM (only results with 0.01< $P \leq 0.05$ are presented here). The Y-axis represents the relative abundance of microbial taxa (arcsine square-root transformation), and the X-axis represents hormone levels ($\log_{10}(x)$ transformation)

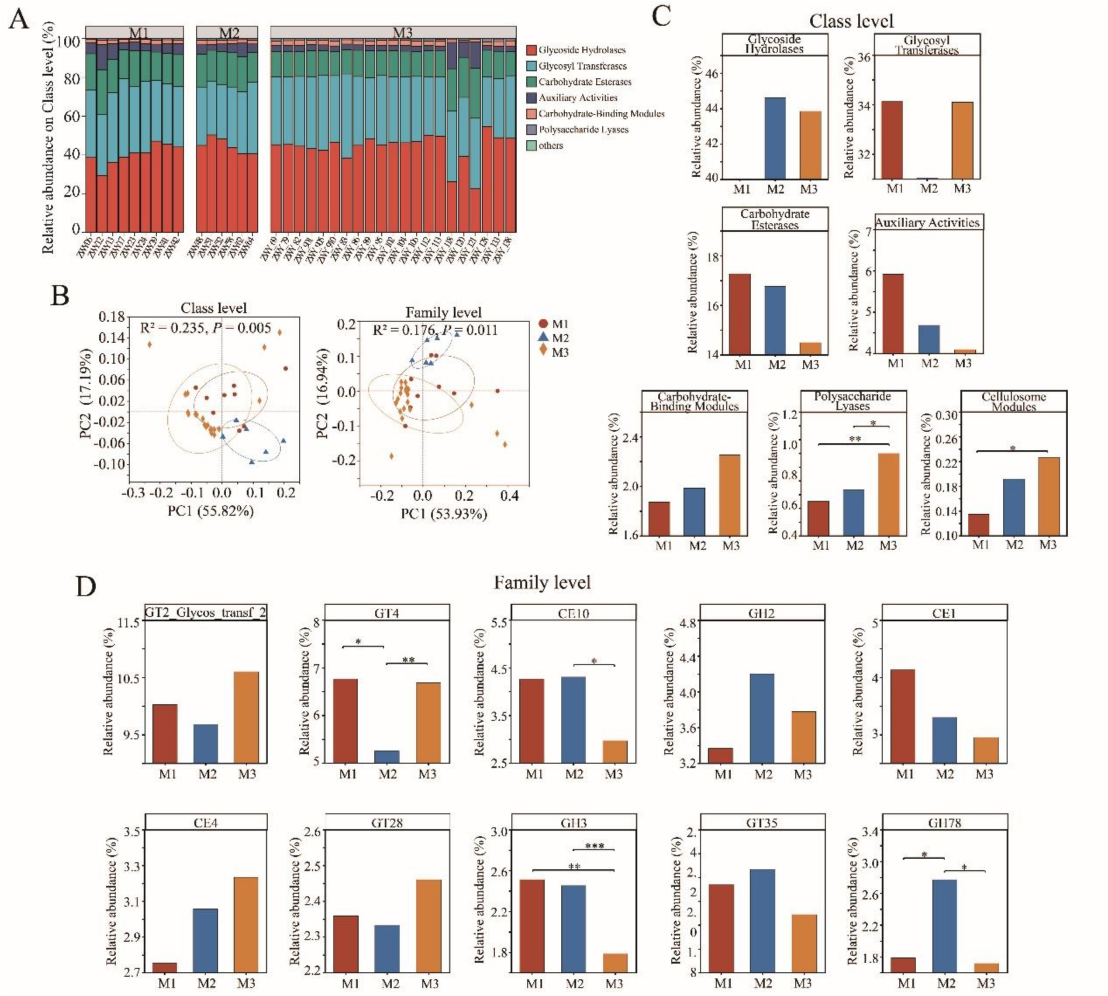

Figure S6. The CAZy genes composition of gut microbiome in different alpha male periods (a) (“others” includes all taxa whose relative abundance is less than 1%), the PCoA results in Class and Family (b) levels, and comparison of CAZy genes Class level (c) and Family level (d) based on GLMM (M1: the period dominated by alpha male 1, M2: the period dominated by alpha male 2, M3: the period dominated by alpha male 3; * for $P < 0.05$, ** for $P < 0.01$, and *** for $P < 0.001$)

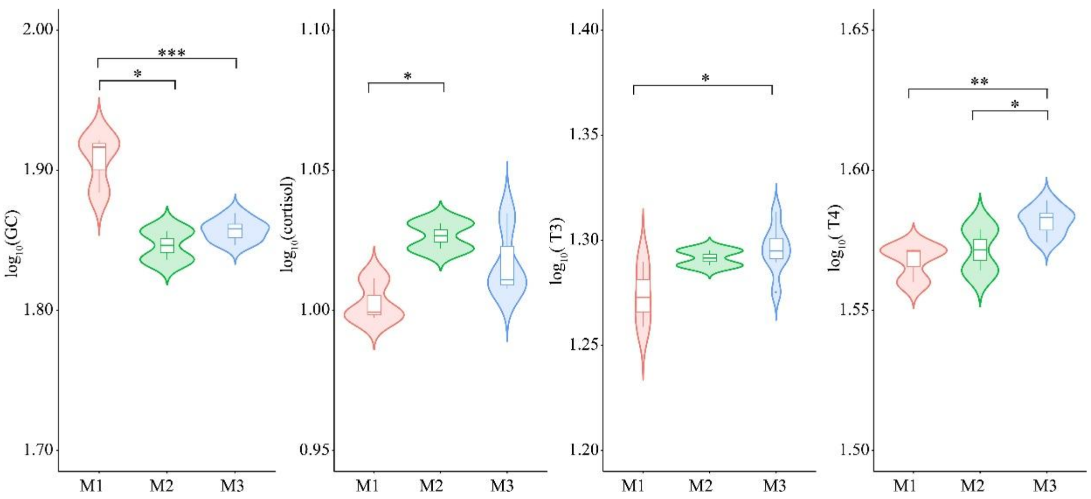

Figure S7. The comparison of hormones among different alpha male periods based on GLMM (M1: the period dominated by alpha male 1, M2: the period dominated by alpha male 2, M3: the period dominated by alpha male 3; * for $P < 0.05$, ** for $P < 0.01$, and *** for $P < 0.001$)

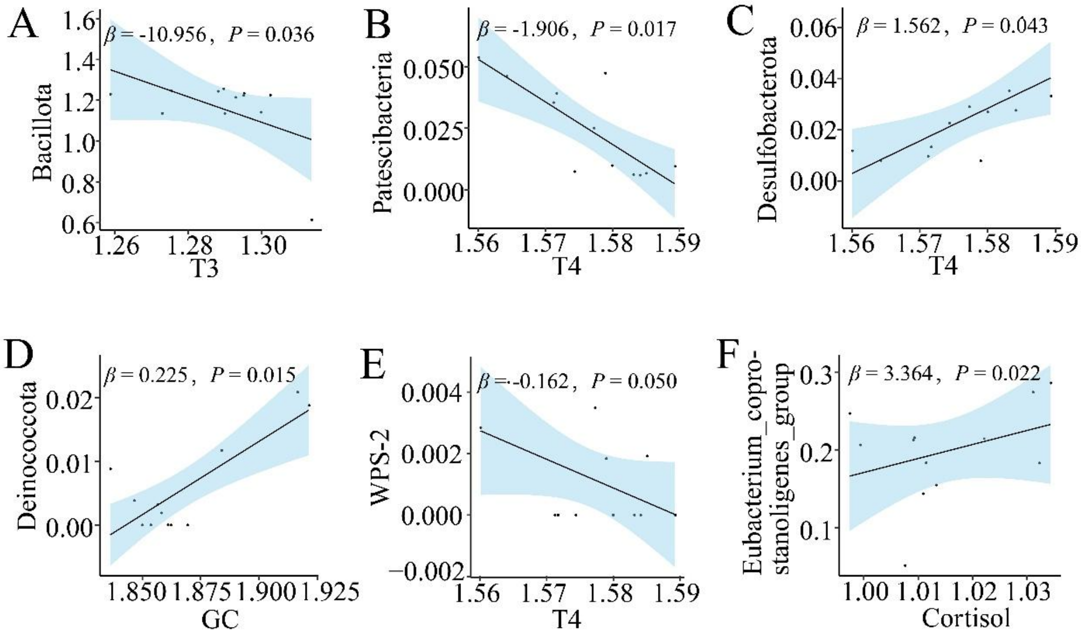

Figure S8. The correlations between hormones and the gut microbiome structure based on the GLM in different alpha male period (only results with 0.01< $P \leq 0.05$ are presented here). The Y-axis represents the relative abundance of microbial taxa (arcsine square-root transformation), and the X-axis represents hormone levels ($\log_{10}(x)$ transformation)

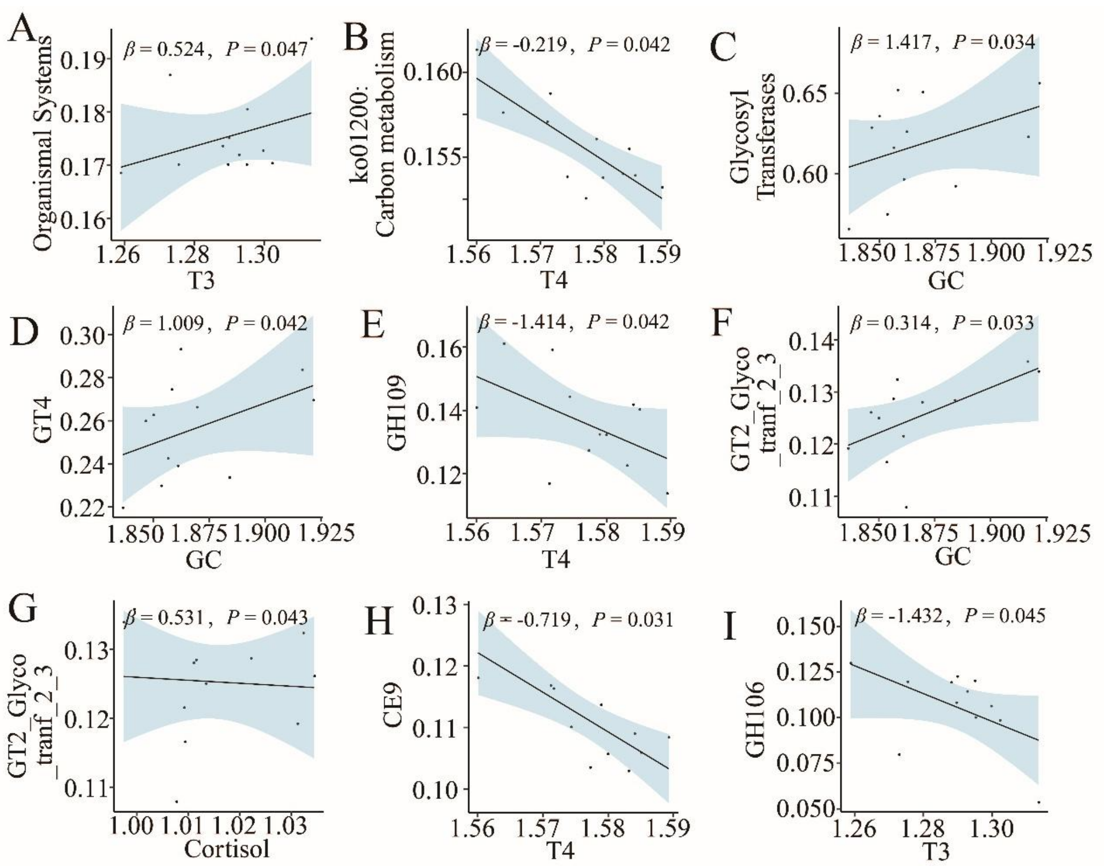

Figure S9. The correlations between hormones and the gut microbiome functional genes based on the GLM in different alpha male period (only results with 0.01< $P \leq 0.05$ are presented here). The Y-axis represents the relative abundance of microbial taxa (arcsine square-root transformation), and the X-axis represents hormone levels ($\log_{10}(x)$ transformation)

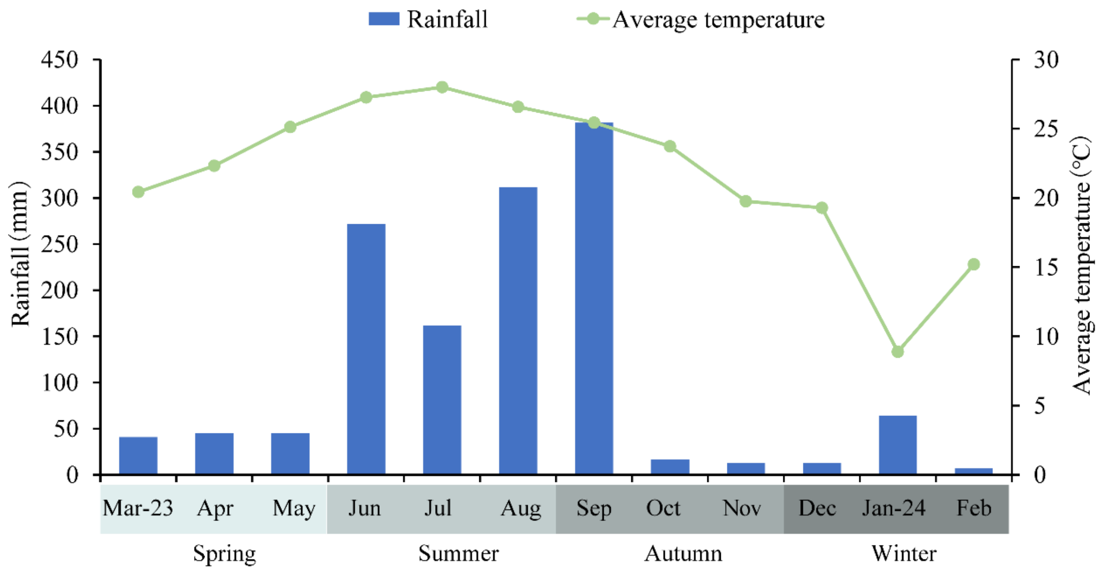

Figure S10. Mean temperature and rainfall in Banli area of the Guangxi Chongzuo White-headed Langur National Nature Reserve
